# The *GALNT9*, *BNC1* and *CCDC8* genes are frequently epigenetically dysregulated in breast tumours that metastasise to the brain

**DOI:** 10.1186/s13148-015-0089-x

**Published:** 2015-05-27

**Authors:** Rajendra P. Pangeni, Prasanna Channathodiyil, David S. Huen, Lawrence W. Eagles, Balraj K. Johal, Dawar Pasha, Natasa Hadjistephanou, Oliver Nevell, Claire L. Davies, Ayobami I. Adewumi, Hamida Khanom, Ikroop S. Samra, Vanessa C. Buzatto, Preethi Chandrasekaran, Thoraia Shinawi, Timothy P. Dawson, Katherine M. Ashton, Charles Davis, Andrew R. Brodbelt, Michael D. Jenkinson, Ivan Bièche, Farida Latif, John L. Darling, Tracy J. Warr, Mark R. Morris

**Affiliations:** Brain Tumour Research Centre, University of Wolverhampton, Wolverhampton, UK; School of Biology, Chemistry and Forensic Sciences, University of Wolverhampton, Wolverhampton, UK; Centre for Rare Diseases and Personalised Medicine, School of Clinical and Experimental Medicine, University of Birmingham, Birmingham, UK; Department of Neurosciences, Lancashire Teaching Hospitals NHS Foundation Trust, Royal Preston Hospital, Fulwood, Preston, UK; The Walton Centre NHS Foundation Trust, Lower Lane, Liverpool, UK; Department of Genetics, Institute Curie, Paris, France

**Keywords:** Breast, Brain, Metastasis, DNA methylation, Epigenetic, Tumour suppressor

## Abstract

**Background:**

Tumour metastasis to the brain is a common and deadly development in certain cancers; 18–30 % of breast tumours metastasise to the brain. The contribution that gene silencing through epigenetic mechanisms plays in these metastatic tumours is not well understood.

**Results:**

We have carried out a bioinformatic screen of genome-wide breast tumour methylation data available at The Cancer Genome Atlas (TCGA) and a broad literature review to identify candidate genes that may contribute to breast to brain metastasis (BBM). This analysis identified 82 candidates. We investigated the methylation status of these genes using Combined Bisulfite and Restriction Analysis (CoBRA) and identified 21 genes frequently methylated in BBM. We have identified three genes, *GALNT9*, *CCDC8* and *BNC1*, that were frequently methylated (55, 73 and 71 %, respectively) and silenced in BBM and infrequently methylated in primary breast tumours. *CCDC8* was commonly methylated in brain metastases and their associated primary tumours whereas *GALNT9* and *BNC1* were methylated and silenced only in brain metastases, but not in the associated primary breast tumours from individual patients. This suggests differing roles for these genes in the evolution of metastatic tumours; *CCDC8* methylation occurs at an early stage of metastatic evolution whereas methylation of *GANLT9* and *BNC1* occurs at a later stage of tumour evolution. Knockdown of these genes by RNAi resulted in a significant increase in the migratory and invasive potential of breast cancer cell lines.

**Conclusions:**

These findings indicate that *GALNT9* (an initiator of O-glycosylation), *CCDC8* (a regulator of microtubule dynamics) and *BNC1* (a transcription factor with a broad range of targets) may play a role in the progression of primary breast tumours to brain metastases. These genes may be useful as prognostic markers and their products may provide novel therapeutic targets.

**Electronic supplementary material:**

The online version of this article (doi:10.1186/s13148-015-0089-x) contains supplementary material, which is available to authorized users.

## Background

Brain metastases account for up to 40 % of all secondary tumours, with an estimated 27,000 new cases every year in the UK [1, 2]. Current estimates suggest that 18–30 % of patients with breast cancer eventually develop brain metastases [3–6]. The frequency of metastatic brain tumours is rising; this increased incidence is due, in part, to an ageing population, improved neuroimaging and increased patient survival following primary tumour treatment [7]. Currently, brain metastases are treated by whole brain radiotherapy, stereotactic radiosurgery and surgical resection either individually or in combination [8]. However, following treatment, patient prognosis remains poor; both morbidity and mortality are high and the median survival is approximately 7 months [9].

Evidence indicating that tumours originating in specific organs favour certain sites of metastasis has existed for over 50 years [10]. However, the underlying mechanisms of this organotropism towards specific secondary sites such as the brain are still poorly understood. Although the genetic basis of primary tumour formation is becoming increasingly clear [11], it is still unclear which of the many hundreds of tumour-associated alterations found in primary breast cancer [12, 13] contribute to metastasis and moreover, metastasis to specific secondary sites such as the brain. The primary tumour types that most frequently metastasise to the brain are lung, breast, melanoma and renal cancers. However, the speed at which these secondary tumours develop varies greatly with breast to brain metastases (BBM) occurring relatively slowly [7]. This specificity indicates that, at least in part, genomic alterations that drive tumour formation in these primary organs provide the potential for colonization of a distinct subset of secondary organ sites.

There is little in the way of prognostic markers for BBM. It is known that the risk of BBM occurring early (<2 years after primary diagnosis) is associated with early onset tumours, estrogen receptor negative (ER-ve), human epidermal growth factor receptor 2 overexpression (HER2 + ve) and triple negative (ER-ve/PR-ve/HER2-ve) phenotypes [14–17]. However, more than 50 % of BBMs occur over 5 years after the primary tumour was diagnosed. Many of these late recurring brain metastases are derived from ER+ primary tumours [4, 9, 18]. The common long lag-time between primary tumour diagnosis and recurrence of a detectable secondary tumour suggests that cells from these breast tumours undergo a period of dormancy [19, 20]. These dormant cells are often found as micrometastases in bone marrow. However, the presence of these micrometastases is not in itself a strong prognostic indicator for later metastatic disease [21, 22]. It is possible that brain micrometastases are common, and these require further genomic alterations to occur before sustained proliferation and growth occurs.

Genomic alterations that provide the potential for metastatic growth can be characterised as either those that also drive primary tumour growth advantage, those that provide potential for dissemination and infiltration [23] or those that enable continued growth within the microenvironment of the new organ [24]. A number of genetic and epigenetic alterations acquired by breast tumour micrometastases of the bone have been characterised [25, 26]. However, very little is known about specific genomic alterations that facilitate colonisation in the brain.

We have carried out a screen to identify genes frequently dysregulated through promoter hypermethylation in BBM. This analysis has identified candidate genes that are either dysregulated early in tumour evolution (methylation is common to primary tumour and resulting BBM) or at a later stage, once the cells that will evolve into the BBM have disseminated from the primary tumour. We hope that this preliminary analysis may provide initial evidence of novel targets that can be utilised in the development of prognostic screens and new rational therapeutic approaches for breast tumours and brain metastases.

## Methods

### Selection of candidate metastatic suppressor genes

For an overview of our candidate selection strategies see Additional file 1: Figure S1. We utilized the Illumina HumanMethylation 450 K BeadChip methylation array data from *The Cancer Genome Atlas* (TCGA) to identify candidate genes (Additional file 2: Table S1 for TCGA tumour barcodes). To ensure we were selecting genuine promoter-associated CpG islands, we selected only those probes that are located in the 5′ region of the gene or up to 1500 base pairs from the transcription start site (identified in the array annotation as TSS, TSS200, TSS1500). We identified individual probes that are not methylated (β value ≤ 0.25) in 75 % (15/20) of primary breast tumours and methylated (β value ≥ 0.60) in primary lung tumours, in at least 50 % (10/20) of the samples. This analysis generated four candidates that were then characterised in the laboratory.

In addition to our bioinformatic analysis, we carried out a broad literature review to identify candidate genes. We generated a long-list of genes that had previously been identified as hypermethylated in one of the primary tumours types that readily metastasise to the brain (lung, melanoma or renal [7]). We expanded this long-list by selecting genes that are downregulated in epithelial-mesenchymal transition (EMT) and that possess a well-defined promoter region CpG island. By interrogating all the available breast tumour methylation data in the TCGA by using their data portal (https://tcga-data.nci.nih.gov/tcga/), we shortlisted only those genes that were infrequently methylated in primary breast tumours. This analysis generated 78 candidates that were then characterised in the laboratory.

### Patients and samples

Thirty-one fresh-frozen metastatic brain tumours originating from primary breast tumours were provided by The Walton Research Tissue Bank, Liverpool and Brain Tumour North West (BTNW) Tissue Bank, Preston. Eleven pairs of formalin fixed paraffin embedded (FFPE) primary breast tumours corresponding to matched metastatic brain tumours were provided by BTNW tissue bank. Receptor status information is available for 9 of the 11 primary tumour pairs, six of these are ER + ve, one is triple negative. The time between primary tumour surgery and removal of the brain metastasis ranges from 2 to 10 years (Additional file 3: Table S2a).

A cohort of 40 independent primary breast tumours was also analysed. All breast tumours from this cohort were ducal carcinomas; their clinical characteristics are described in [27]. Molecular characterisation was available for 20 of these tumours, 15 of these are ER + ve and three are triple negative. No brain metastases were observed in any of these patients, nine patients had been screened for metastasis 10 years or more post-primary tumour surgery and 17 of the 20 after more than 5 years (Additional file 3: Table S2b).

Tissues were obtained from National research Ethics committee approved research tissue banks, and informed consent was obtained from each patient. This study was conducted according to the principles expressed in the Declaration of Helsinki.

### Breast cancer cell Lines and 5-Aza-2′–deoxycytidine treatment

Five breast cancer cell lines (MCF7, T74D, MDA-MB231, BT549 and ZR75) were routinely maintained in DMEM (Sigma, UK) supplemented with 10 % FCS at 37 °C and 5 % CO_2_. Cells were plated according to their doubling time to ensure that both control and 5-AZA-2′-deoxycytidine (5-AZA-dC; Sigma, UK)-treated cells lines were approximately 75 % confluent at the time of RNA extraction. 5-AZA-dC was freshly prepared in ddH_2_O and filter sterilized. Twenty-four hours after seeding, cells were treated with 5 μM 5-AZA-dC. Cells were treated with fresh 5 μM 5-AZA-dC three times a week on alternate days. After 7 days, the cells were collected using 1 % trypsin; cell pellets were washed with PBS.

### Genomic DNA/RNA extraction

Genomic DNA was extracted from fresh-frozen metastatic brain tumours using The *DNA isolation kit from cells and tissues* (Roche, Germany). Briefly, 25 mg of tissue was homogenised using lysis buffer and incubated at 37 °C for 30 min followed by addition of Proteinase K and RNase solution. The samples were then centrifuged and processed according to manufacturer’s instructions. For FFPE samples, a *FFPE DNA extraction kit* (Qiagen, USA) was used. Briefly, a small block of samples embedded with paraffin was cut into thin sections and mixed with xylene followed by 100 % ethanol. The samples were then processed according to manufacturer’s instructions. Similarly, total RNA was extracted using the *EZ-RNA extraction kit* (Biological Industries, Israel). Briefly, fresh-frozen tumours were homogenized using lysis buffer followed by addition of extraction solution. The samples were then centrifuged and processed according to manufacturer’s instructions. DNA concentration was measured using a *nanodrop2000* (Thermo Scientific, USA).

### Bisulfite conversion of DNA

Bisulfite conversion of genomic DNA from metastatic brain tumours (500 ng) and positive controls was carried out using the *EZ DNA methylation kit* (Zymo Research Corp., USA) according to manufacturer’s instruction. Fully methylated, positive controls were generated by incubating gDNA with DNA methyltransferase, in the presence of S-Adenosyl methionine (SAM) (New England bio lab, USA) for 2 h at 37 °C prior to bisulfite conversion.

### Promoter methylation analysis

Primers used to amplify promoter regions from bisulfite-modified DNA can be found in Additional file 4: Table S3. Primers were designed based on standard bisulfite DNA primer designing criteria [28]. These primers were used to amplify bisulfite converted DNA. DNA methylation was determined by digesting Combined Bisulfite and Restriction Analysis (CoBRA) PCR products with the BstUI and TaqI restriction enzymes (Fementas, UK).

Quantitative methylation analysis of tumour DNA was carried out by cloning bisulfite-PCR products (individual alleles) into pGEM plasmid (Promega, UK) followed by sequencing of individual clones using primers to M13.

The CpG island regions of *BNC1*, *CCDC8* and *GALNT9* are presented in Additional file 5: Figure S2, details of PCR primer sites and individual CpG dinucleotides analysed by sequencing are provided.

### Migration assay

Candidate genes were knocked down in breast cancer cell lines by transfection of RNAi ‘silencer select’ oligos against *CCDC8* (s228331), *BNC1* (s2012) or *GALNT9* (s27040), control cells were transfected with control oligo no. 1 (Ambion, Austin, TX, USA). After 24 h, DMEM with 10 % FBS was replaced with fresh DMEM without FBS and incubated at 37 °C for 24 h. Confluent monolayer of cells in each well was scratched with the tip of a 200 μl pipette tip. The extent of migration of cells was observed after 24 and 48 h.

### Invasion assay

Two hundred microlitres of matrigel matrix (Becton Dickinson, NJ, USA) was applied to 24-well 9-mm inserts containing polyethylene terephthalate (PET) membranes with 8-um pores (Corning, USA). One hundred fifty thousand cells were applied to the invasion chamber. DMEM containing 10 % FBS was placed in the lower chamber as a chemoattractant. The plates were incubated at 37 °C for 48 h with 5 % CO_2_. Cells from the lower layer were stained with crystal violet. The optical density at 540 nm for each well was determined.

### Western blotting

Cells were lysed in RIPA buffer (25 mM HCL, 0.1 % SDS, 1 % triton 100, 0.15 M NaCl) containing phosphatase and protease inhibitor (Roche, Germany). Seventy micrograms of each extract was resolved on polyacrylamide gels and probed with anti-rabbit primary antibody against *CCDC8* (ab170233), *BNC1* (ab123645) or *GALNT9* (ab173682) (Abcam, USA). Signals were detected with horseradish peroxidase-conjugated anti-rabbit antibody (GE Healthcare, UK) and enhanced chemiluminescence (Biological Industries, Israel). Membranes were stained with India ink (Winsor and Newton, UK) for comparison of loading.

## Results

### Screening of candidate BBM suppressor genes

We have used a candidate gene approach to identify genes deregulated in breast tumours that metastasise to the brain. See Additional file 6: Table S4 for details of genes.

### Bioinformatic analysis of primary tumour genome-wide methylation arrays identified four candidate BBM suppressor genes

We have compared the methylation status (β value) of array probes in *TCGA* data sets from 20 primary breast tumours (with no evidence of metastatic disease) and 20 primary lung tumours. We hypothesised that genes that are infrequently methylated in non-metastasising breast tumours and frequently methylated in primary lung tumours that readily metastasis to the brain (metastases are identified relatively soon after primary tumour diagnosis) [29] may be commonly methylated in metastatic brain tumours that derive from both lung and breast tumours.

We filtered probes in primary breast tumours to identify those that are infrequently methylated, (having a β value ≤ 0.25 in at least 15/20 (75 %)). This resulted in 97,155 probes. Filtering of frequently methylated probes, (having a β value ≥ 0.60 in at least 50 % (10/20)) in lung tumours resulted in 45,382 probes. Comparison of the probes between breast and lung tumours identified eight probes that corresponded to six genes (*GALNT9*, *KRT222*, *PLEKHA6*, *TFAP2A*, *TSPAN4* and *ZNF808*). Two of these genes (*KRT222* and *PLEKHA6*) do not have well-defined CpG islands. In total, this genome wide approach identified four candidate genes (*GALNT9*, *TFAP2A*, *TSPAN4* and *ZNF808*) for further analysis.

### A literature review identified 78 candidates BBM suppressor genes

We have screened genes that have previously been shown to be frequently methylated and silenced in at least one of the primary tumours types that rapidly (relative to many breast tumours) metastasise to the brain, i.e. lung, melanoma and renal cell carcinoma (RCC) [7]. We then interrogated *TCGA* to determine the methylation status of these genes in primary breast tumours. This screen identified 42 candidate metastatic suppressor genes that are infrequently methylated in primary breast cancer and frequently methylated in primary lung, melanoma or renal tumours (Additional file 6: Table S4 and references therein).

In addition, we selected 36 metastasis suppressor candidates that are downregulated during EMT (Additional file 6: Table S4 and references therein).

### Identification of frequently methylated genes in metastatic brain tumours

The methylation status of 82 candidate genes was determined by CoBRA [28] in 30 BBM. To ensure that we were identifying genes which are enriched in the population of patients with BBM that are most likely to be clinically significant, we have imposed a high cut-off of ≥50 % of all metastatic tumours being methylated for a gene to be considered as frequently methylated. For this preliminary screening, we have determined that a significant proportion of the promoters within the tumour sample is methylated if there are clearly observed digest products following restriction analysis.

**Fig. 1:**
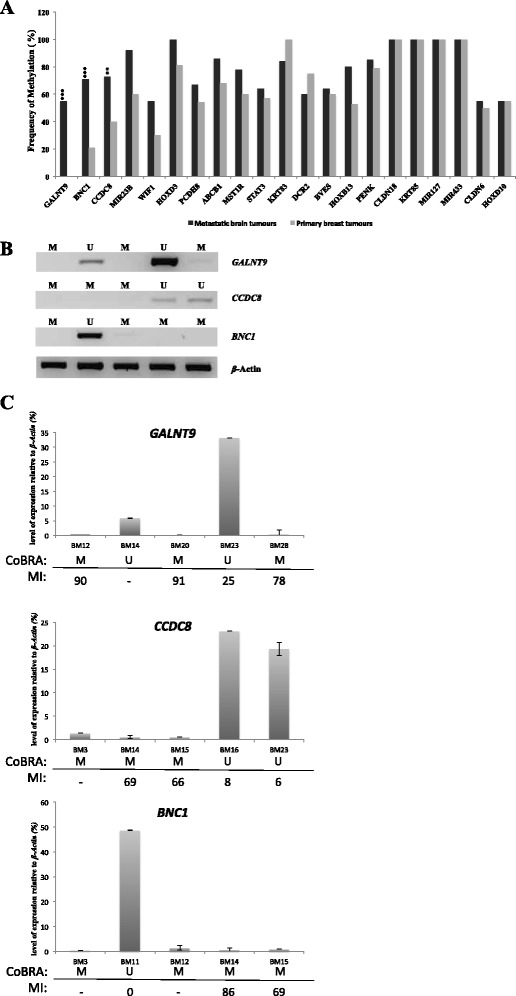
**a** Methylation frequency of candidate metastatic suppressor genes in breast-to-brain metastases (BBM) (*n* = 30) versus primary breast tumours (*n* = 40). Out of the 25 genes that were frequently methylated in brain metastases, three genes (*GALNT9*, *CCDC8* and *BNC1*) were infrequently methylated in a cohort of independent primary tumours with statistical significance (*p* = 0.0001, 0.01 and 0.0001, respectively). **b**, **c** Expression of *GALNT9*, *CCDC8* and *BNC1* correlates with promoter methylation in BBM. Reverse transcription PCR (RT-PCR) for *GALNT9*, *CCDC8* and *BNC1* in BBM shows that these genes were expressed in tumours where their promoters are unmethylated (U) and silenced in methylated (M) tumours (see Additional file 7: Figs S3 and Additional file 9: Figure S5 for representative methylation analysis). Expression of β-actin was determined to ensure equal loading for all samples. **c** Expression levels of each gene were quantified in relation to the expression of β-actin. The methylation status was determined by either CoBRA or sequencing of individual alleles to determine the methylation index (MI) for individual tumours. High levels of expression were not associated with high levels of methylation in the region analysed. A full set of methylation/expression analysis can be seen in Additional file 10: Figure S6 (*BM* brain metastasis, *M* methylated, *U* unmethylated, − analysis was not done)

From the panel of four genes selected from our analyses of HumanMethylation 450 K BeadChip arrays obtained from TCGA, only one gene, *GALNT9*, was frequently methylated (55 %) in metastatic brain tumours originating from primary breast tumours (see Table 1, Fig. 1a, Additional file 7: Figure S3).

**Table 1:** Genes frequently methylated in breast to brain metastases. Twenty-one genes are frequently methylated in brain metastases (n = 15) of which 18 genes are also frequently methylated in primary breast tumours (n = 20). Three genes, CCDC8, BNC1 and GALNT9 (highlighted in grey), are infrequently methylated in primary breast tumours. These genes were further analysed in 20 primary breast samples (n = 40 in total) and 15 breast to brain metastases (n = 30 in total)

Gene symbol	Accession	Gene name	% of metastatic tumours methylated	Function
*CLDN18*	NM_016369.3	*Claudin 18*	100	Intercellular adhesion molecule responsible for tight junction strand formation [77]
*KRT85*	NM_002283.3	*Keratin 85*	100	Component of intermediate filament in epithelial cells contributing to cell-cell adhesion [78–80]
*MIR127*	NR_029696.1	*microRNA 127*	100	Regulator of cell proliferation and senescence [81]
*MIR433*	NR_029966.1	*microRNA 433*	100	Deregulated in gastric cancer, regulator of cell migration and drug response [82, 83]
*HOXD3*	NM_006898.4	*HomeoboxD3*	100	Proangiogenic transcription factor [84]
*MIR23B*	NR_029664.1	*microRNA 23b*	92	Involved in cytoskeleton modelling, motility and metastasis [85–88]
*CCDC8*	NM_032040.4	*Coil coiled domain containing 8*	73	Mutated in patients with 3 M syndrome [70]. Loss is associated with genomic instability and aneuploidy [75].
*KRT83*	NM_002282.3	*Keratin 83*	84	Component of intermediate filament, contributes to cell to cell adhesion [78, 80]
*HOXB13*	NM_006361.5	*Homeobox B13*	80	TSG for prostate cancer, inhibits androgen mediated signalling [89]
*ABCB1*	NM_000927.4	*ATP-binding cassette sub-family B member 1*	80	Controls efflux of substances across plasma membranes, associated with multidrug resistance [90]
*PENK*	NM_006211.3	*Proenkephalin*	80	Promotes RNA splicing in osteoblasts and neural cells, plays role in bone development [91]
*MST1R*	NM_002447.2	*Macrophage stimulating 1 receptor*	78	Involved in intracellular signalling cascades leading to cellular growth, motility and invasion [92]
*BNC1*	NM_001717.3	*Basonuclin 1*	71	Zink finger transcription factor, regulator of EMT [68]
*PCDH8*	NM_002590.3	*Procadhern 8*	73	Helps in cell to cell adhesion [93]
*STAT3*	NM_139276.2	*Signal transducer and activator of transcription 3*	67	Involved in embryonic stem cell regulation, somatic cell growth [94–96]
*BVES*	NM_007073.4	*Blood vessel epicardial substance*	64	Involved in inter-cellular interaction and cell adhesion [97]
*TNFRSF10D*	NM_003840.4	*Tumour Necrosis Factor receptor superfamily 10 D*	60	Member of TNF (Tumour Necrosis Factor) receptor superfamily, promotes apoptosis in cancer cells [98]
*CLDN6*	NM_021195.4	*Claudin 6*	55	Intercellular adhesion molecules responsible for tight junction strand formation, its epigenetic silencing is associated with migration and invasiveness of breast cancer [77, 99]
*HOXD10*	NM_002148.3	*Homeobox D10*	55	Maintain epithelial cell plasticity and contributes to stability of extracellular matrix [100]
GALNT9	NM_001122636.1	*N-acetyl galactosaminyl transferase 9*	55	Catalyzes O-glycosylation [53, 101]
*WIF1*	NM_007191.4	*Wnt inhibitory factor-1 gene*	53	Inhibitor of Wnt-signalling [102, 103]

From our panel of 42 literature review candidate genes, we identified ten genes that are frequently methylated in brain metastases. These were *HOXD3* (100 %), *CCDC8* (73 %), *HOXB13* (80 %), *ABCB1* (80 %), *PENK* (80 %), *BNC1* (71 %), *PCDH8* (53 %), *STAT3* (67 %), *TNFRSF10D* (60 %) and *WIF1* (53 %) (see Table 1, Fig. 1a, Additional file 7: Figure S3).

We proceeded to determine the methylation status of these ten genes in an independent cohort of primary breast tumours.

In addition, from a panel of 36 genes downregulated in EMT, we identified 10 genes frequently methylated in metastatic brain tumours originated from primary breast tumours. These were *CLDN18* (100 %), *KRT85* (100 %), *MIR127* (100 %), *MIR433* (100 %), *MIR23b* (92 %), *KRT83* (84 %), *MST1R* (78 %), *BVES* (64 %), *CLDN6* (55 %) and *HOXD10* (55 %) (see Table 1, Fig. 1a). We proceeded to determine the methylation status of these ten genes in an independent cohort of primary breast tumours.

A graphical overview of our candidate selection process and results is presented in Additional file 1: Figure S1.

### *GALNT9*, *BNC1* and *CCDC8* are differentially methylated in primary breast tumours and BBM

We have screened primary breast tumours for the presence of methylation in the genes that are frequently methylated in BBM. To ensure that genes identified in this study are clinically significant, we have imposed a relatively low cut-off frequency of ≤45 % for methylation in primary breast tumours.

We analysed the 21 genes that were frequently methylated in BBM in a cohort of 40 primary breast tumours (unrelated to the brain metastasis cohort [27]).

We found *GALNT9* to be frequently methylated in BBM (55 %) and not methylated in any of the 40 primary breast tumours (*p* = 0.0001).

From a panel of ten genes frequently methylated in brain metastases (from our literature review candidates), we identified that eight of these genes were also frequently methylated in primary breast tumours. These are *HOXD3* (81 %), *HOXB13* (53 %), *ABCB1* (68 %), *PCDH8* (54 %), *PENK* (79 %), *STAT3* (57 %), *TNFRSF10D* (75 %) and *WIFI* (55 %) (Fig. 1a). This suggests that these genes are not uniquely epigenetically deregulated during the process of BBM. However, it is worth noting that to our knowledge this is the first time that promoter methylation in *CCDC8*, *HOXD3*, *PCDH8*, *PENK*, *STAT3*, *SFRP2* and *WIFI* has been described in primary breast tumours.

Promoter methylation of *BNC1* (17 %) and *CCDC8 (*40 %) in primary breast tumours was infrequent (≤45 %), and statistically significantly lower than that of the frequency of methylation in BBM (*p* = 0.0001 and 0.01, respectively) (Fig. 1a). The low frequency of methylation in primary tumours indicates that *BNC1* and *CCDC8* may contribute to BBM and are good candidates for further investigation.

We found that all ten EMT-related genes were frequently methylated in primary breast tumours, i.e. *CLDN18* (100 %), *KRT85* (100 %), *MIR127* (100 %), *MIR433* (100 %), *MIR23b* (60 %), *KRT83* (100 %), *MST1R* (60 %), *BVES* (60 %), *CLDN6* (50 %) and *HOXD10* (55 %) (Fig. 1a). The high frequency of methylation in primary tumours indicates that epigenetic deregulation of these genes is not driving BBM.

From our broad-ranging screens, we have identified *GALNT9*, *BNC1* and *CCDC8* as frequently methylated in BBM and significantly less frequently methylated in primary breast tumours (Fig. 1, Additional file 8: Figure S4).

To ensure that CoBRA digests were representative of high methylation status in tumours, we carried out base-resolution analysis of promoter region methylation for *BNC1*, *CCDC8* and *GALNT9* by cloning and sequencing individual bisulfite-modified alleles from select tumours (Additional file 9: Figure S5). This analysis was used to determine the methylation index (MI) of CpG islands for individual tumours. MI is defined as the total number of methylated CpG dinucleotides given as a percentage of all CpGs analysed. The MI for regions determined to be methylated by CoBRA ranged from 60 to 91 % whereas those promoters deemed not to be methylated by CoBRA had MIs ranging between 0 and 36 %. From this analysis, we have defined that, for these samples, physiologically significant methylation levels are those of ≥60 % MI and lack of physiologically significant methylation is defined as <40 % MI.

### Expression analysis of *BNC1*, *CCDC8* and *GALNT9* in metastatic brain tumours

Having identified three candidate genes that are differentially methylated in primary breast tumours and metastatic brain tumours, we proceeded to determine if this promoter methylation correlated to gene expression.

Total RNA was extracted from 15 metastatic brain tumours to determine the expression of *BNC1*, *CCDC8* and *GALNT9* by RT-PCR. The expression level of each gene was quantified in relation to the expression of β-actin, in tumours with unmethylated promoters (MI = 0–25 %). The maximum expression of these genes was 49, 23 and 33 % that of β-actin, respectively. *BNC1*, *CCDC8* and *GALNT9* were frequently downregulated or silenced in these tumours and reduced expression correlated to promoter methylation as determined by CoBRA and base-resolution sequencing (Fig. 1b and c, Additional file 10: Figure S6). These genes were also commonly silenced in breast cancer cell lines, this silencing was reversed following treatment with 5-Aza-2′-deoxycytidine an inhibitor of DNA methyltransferase enzymes [30] (Additional file 11: Figure S7).

### Promoter methylation status of *BNC1*, *CCDC8* and *GALNT9* in brain metastases and associated primary breast tumours from individual patients

**Fig. 2:**
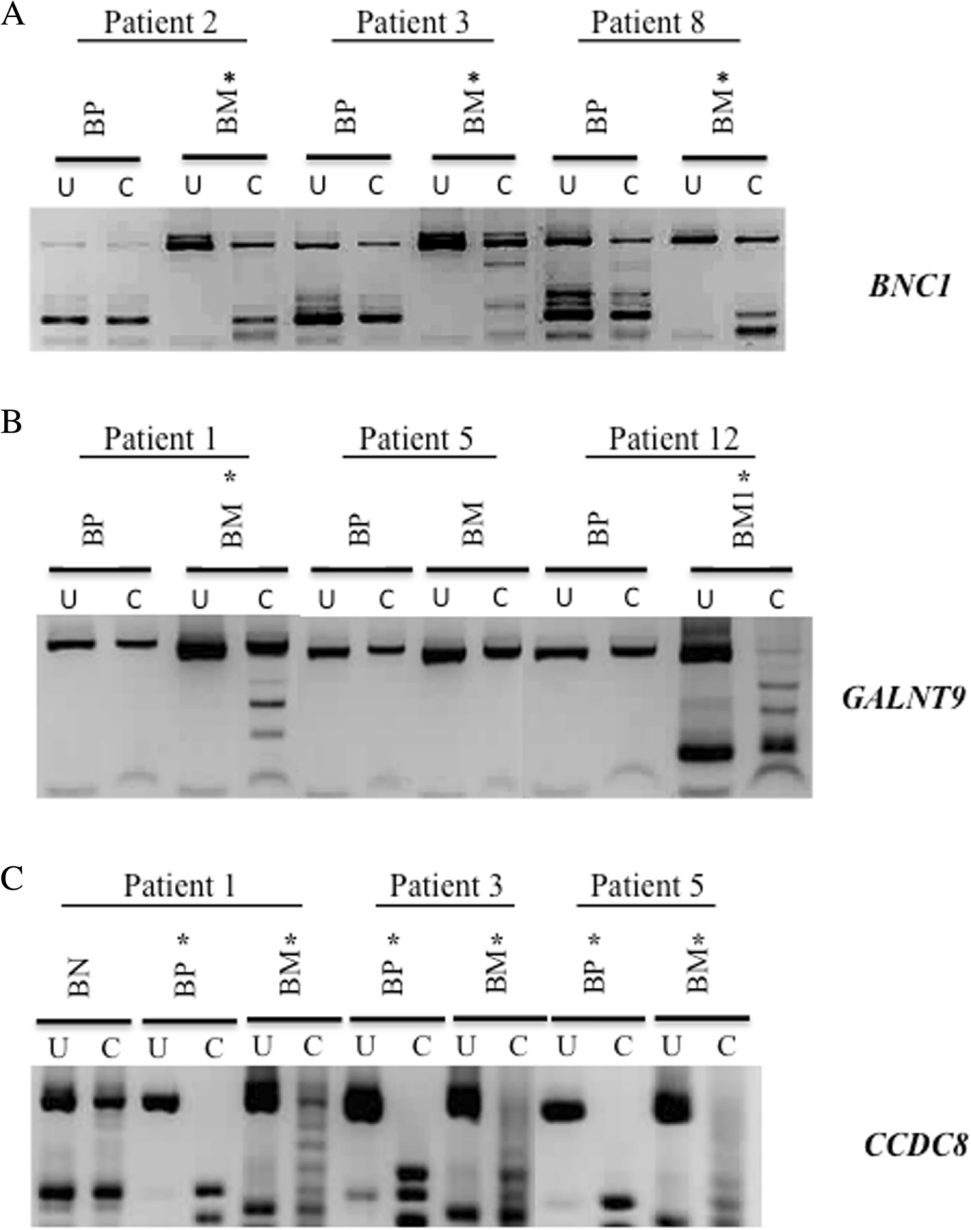
Methylation status of *GALNT9*, *CCDC8* and *BNC1* in metastatic brain tumours and their corresponding originating primary breast tumours from individual patients. CoBRA was used to determine methylation status; small, digested PCR products in the Bstu1 cut (C) lane compared to the undigested (U) lane indicates promoter methylation in a sample. **a** *GALNT9*, **b** *CCDC8* and **c** *BNC1* were frequently methylated (*) in metastatic brain tumours (BM). However, *GALNT9* and *BNC1* were not commonly methylated in the originating breast primary (BP) tumours (**a**, **c**). *CCDC8* promoter was methylated in both the originating primary tumours (BP) and the associated brain metastases (BM) from individual patients (**b**). Of eight matched pairs analysed, *BNC1* was methylated in all metastatic brain tumours whereas it was methylated in only one of the corresponding primary tumours (for example, see patients 2, 3 and 8). Of six matched pairs analysed, *GALNT9* was methylated in three metastatic brain tumours (see patients 1 and 12), whereas it was not methylated in any of the corresponding primary tumours. Of 11 matched pairs analysed, *CCDC8* was methylated in ten metastatic tumours and all corresponding primary tumours (for example, see patients 1, 3 and 5). However, it was not methylated in normal tissue (BN) adjacent to the primary breast tumour (see patient 1). (*BP* breast primary tumour, *BM* metastatic brain tumour, *BN* adjacent normal breast tissue, *U* uncut/control sample, *C* cut by methylation specific restriction enzyme, *methylated samples)

We analysed the methylation status *BNC1*, *CCDC8* and *GALNT9* in metastatic brain tumours and corresponding primary tumours from individual patients. We had ten pairs, however, some loci in the primary tumour DNA proved refractive to amplification. Of eight matched pairs, where the *BNC1* promoter region was successfully amplified, the region was methylated in all eight of the brain metastases. However, it was only methylated in one corresponding primary tumour (Fig. 2a). The *GALNT9* promoter was methylated in 3/5 brain metastases and not methylated in any of the corresponding primary breast tumours (Fig. 2b). In contrast, out of 11 matched pairs, *CCDC8* was commonly methylated in 10 corresponding primary tumours (Fig. 2c). This common *CCDC8* methylation in primary breast tumour and resulting brain metastasis was confirmed by sequencing individual alleles for pairs of tumours from two patients (patient 11 and 15 (BM11, Primary BT 11 and BM15, Primary BT 15)). Both primary tumour DNA and BM DNA were found to have MIs above 73 % (Additional file 9: Figure S5a).

These results suggest that *BNC1* and *GALNT9* promoter methylation occurs at a late stage in the evolution of metastatic brain tumours, possibly after they have metastasised to the brain. Alternatively, methylation of these genes may occur in a small subset of cells within the primary tumour (below the detection threshold of this assay), and these cells are enriched in the metastatic tumour. In contrast, *CCDC8* promoter methylation is detectable in most primary tumours that metastasise to the brain, suggesting that it may play an important role in the early stages of primary tumour metastasis.

### Loss of *GALNT9*, *CCDC8* or *BNC1* expression increases metastatic potential

We have identified that *CCDC8* is dysregulated at an early point of BBM, and its promoter methylation is detectable in the primary tumours that proceed to metastases. *GALNT9* and *BNC1* methylation is uncommon in primary breast tumours and is often not detectable in the tumours that metastasise. These differences suggest that loss of these genes confers metastatic potential though alternative mechanisms. However, loss of *BNC1* or *CCDC8* expression has previously been shown to increase the clonagenic potential of RCC cell lines [31, 32]. Loss of *GALNT9* has yet to be directly linked with increased malignancy. We have investigated the effect that loss of these genes has on metastasis-related properties of breast cancer cell lines.

### Loss of *GALNT9*, *CCDC8* or *BNC1* expression increases breast cancer cell line cell motility

Forty-eight hours after initial transfection with siRNA oligos against *BNC1*, *CCDC8* or *GALNT9* breast cancer cell lines showed loss of specific gene expression (Additional file 12: Figure S8).

**Fig. 3:**
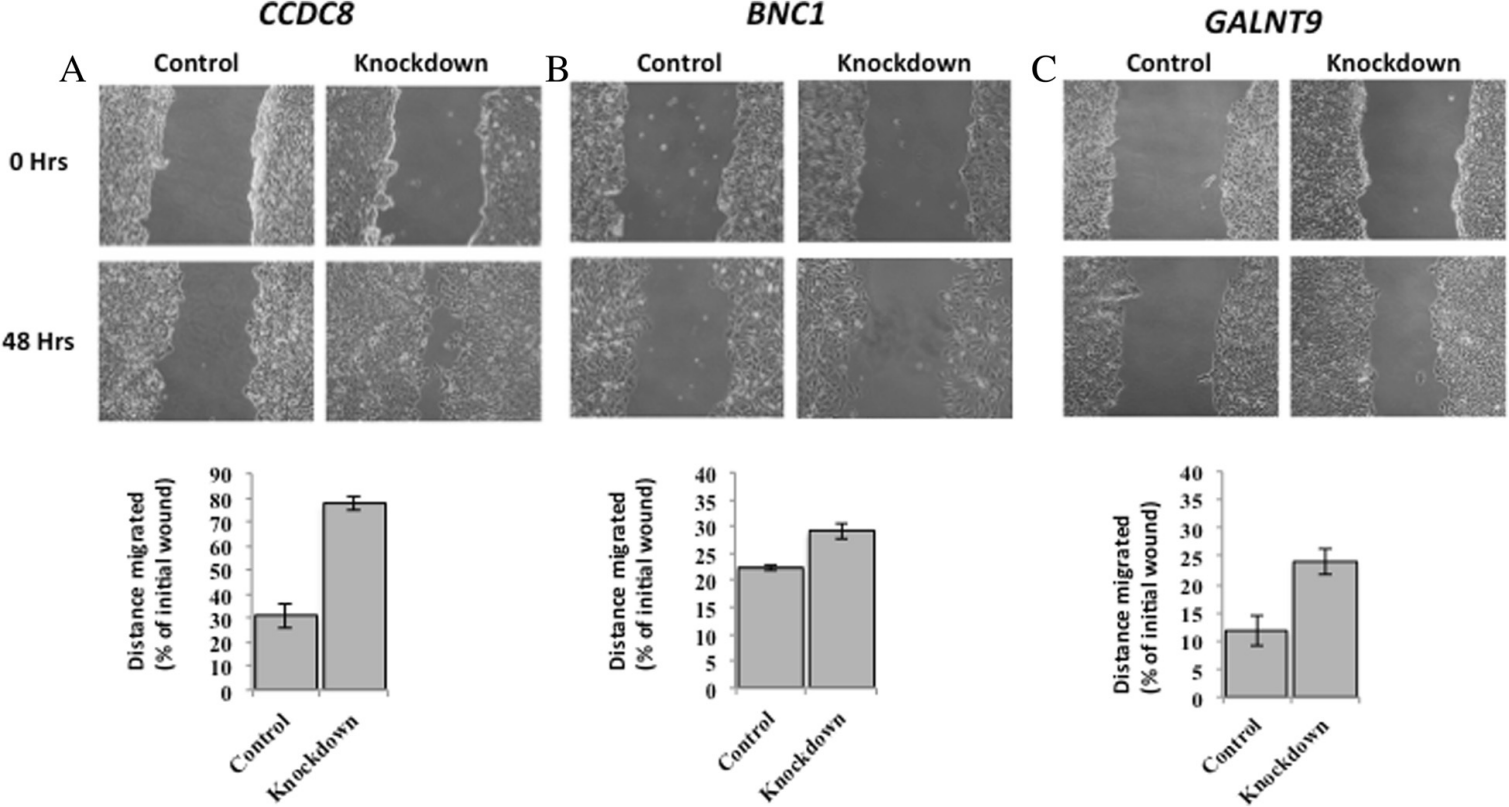
Loss of *CCDC8*, *BNC1* or *GALNT9* expression increases the migratory potential of breast cancer cell lines. Breast cancer cell lines that expressed *CCDC8*, *BNC1* or *GALNT9* were identified; the expression of these genes was knocked down by siRNA (see Additional file 11: Figure S7 and Additonal file 12: Fig. S8). **a** T47D cell lines transfected with siRNA oligos against *CCDC8*; **b** MCF7 cell lines transfected with siRNA oligos against *BNC1* or **c** MDA-MB231 cell lines transfected with siRNA against *GALNT9* exhibited more migratory potential compared to respective cell lines transfected with control siRNA oligos. Following siRNA transfection, confluent cells were incubated in serum-free media and an artificial wound was scratched through them (0 h). Forty eight hours later the distance migrated was calculated by subtracting the value of non-migrated distance from the initial would. The distance migrated (in percentage) by respective cell lines knocked down with siRNA against *CCDC8*, *BNC1* or *GALNT9* in compared to the respective cell lines transfected with control siRNA oligos was statistically significant (*p* = 0.001, 0.011 and 0.027, respectively)

In a wound-healing assay, knockdown of these genes increased migratory potential compared to cell lines transfected with control oligos. The increase in motility of cell lines following knockdown of *BNC1* (Fig. 3a), *CCDC8* (Fig. 3b) or *GALNT9* (Fig. 3c) was statistically significant compared to control cells (scrambled siRNA transfected) (BNC1, *p* = 0.011; *CCDC8*, *p* = 0.001; *GALNT9*, *p* = 0.027). All experiments were repeated in triplicate.

### Reduced expression of *GALNT9*, *CCDC8* or *BNC1* increases invasive potential

*GALNT9*, *BNC1* and *CCDC8* were knocked down in breast cancer cell lines by siRNA and applied to matrigel-coated invasion chambers as described in the methods. Forty-eight hours later, cells that had ‘invaded’ were isolated and quantified.

**Fig. 4:**
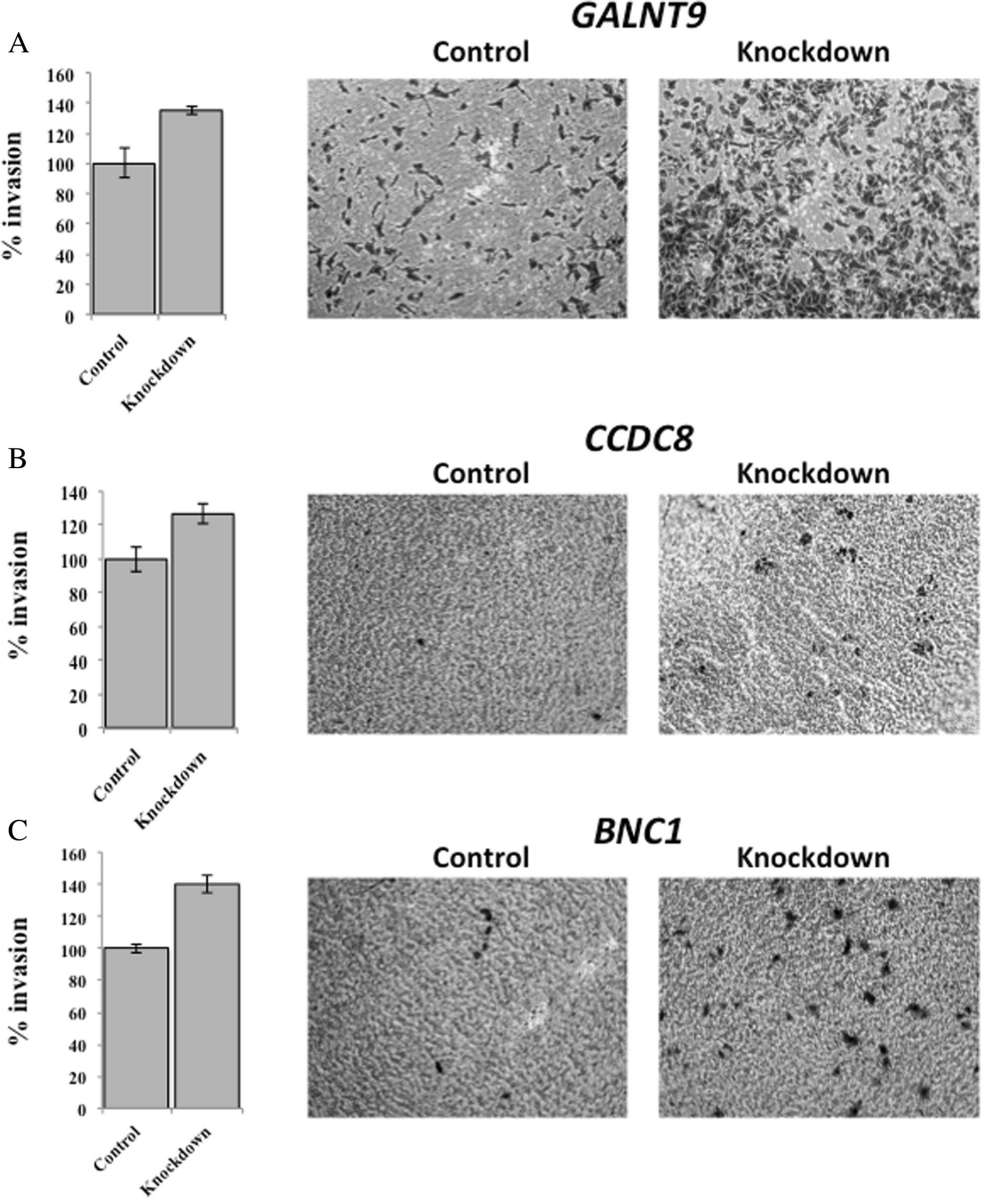
Reduced expression of *GALNT9*, *CCDC8* or *BNC1* increases the invasive potential of breast cancer cell lines. Trans-well invasion assays were carried out following the knockdown of of *GALNT9*, *CCDC8* or *BNC1* in breast cancer cell lines. The invasive capacity of these cells was compared with the same cell lines transfected with control siRNA oligos (control). The numbers of cells that had invaded a matrigel-coated micropore membrane was determined colourimetrically 48 h after initial seeding. **a** MDA-MB231 cell lines transfected with siRNA oligos against *GALNT9*, **b** T47D cell lines transfected with siRNA oligos against *CCDC8* and **c** MCF7 cell lines transfected with siRNA oligos against *BNC1* exhibited a statistically significant increase in invasiveness compared to negative control siRNA transfected cells. *p* = 0.025 (*GALNT9*), *p* = 0.021 (*CCDC8*) and *p* = 0.001 (*BNC1*). Invasive potential was calculated as a percentage increase above that observed for the control cells (% invasion)

Following knockdown of *GALNT9*, 35 % more cells invaded (*p* = 0.025) compared to cell transfected with the control scrambled siRNA (Fig. 4a). Following knockdown of *CCDC8*, 27 % more cells invaded, (*p* = 0.021) (Fig. 4b). The number of breast cancer cell that invaded following *BNC1* knocked down was increased by 40 % (*p* = 0.006) (Fig. 4c).

Increased motility and invasive potential following reduction of expression of these genes suggests that these candidates may be involved in the regulation of normal cellular physiology and that loss of their expression may contribute the metastatic process.

### Reduced expression of *GALNT9* or *CCDC8* is significantly associated with poor relapse-free survival

**Fig. 5:**
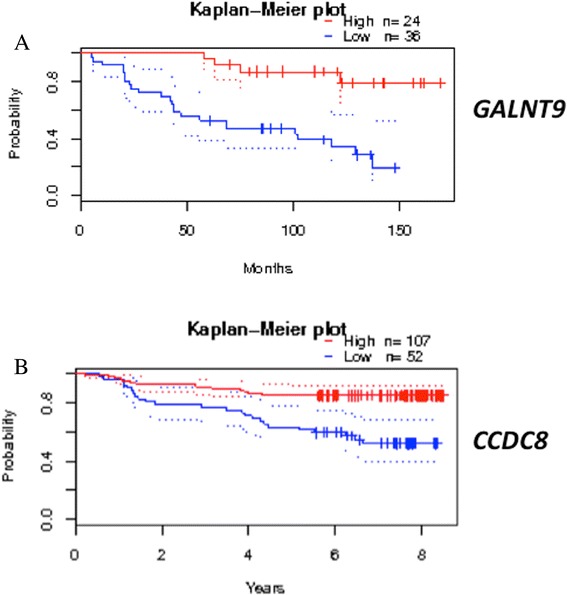
Loss of expression of *CCDC8* and *GALNT9* correlates with relapse-free patient survival. Kaplan–Meier analysis of multiple gene expression studies via the prognoscan database revealed that in two separate studies low expression of **a** *CCDC8* (*p* = 0.001) and **b** *GALNT9* (*p* = 0.003) was associated with poor relapse free survival

The clinical significance of the expression of *BNC1*, *CCDC8* and *GALNT9* was analysed using publically available GEO expression profiles using the prognoscan database [33]*.* Prognoscan partitions a patient population into high-expressor and low-expressor group for each gene by choosing a threshold that maximises the statistical significance of difference in outcome. It corrects for multiple testing using the method of Miller and Siegmund [34]. In two independent datasets, low *CCDC8* expression was significantly associated with poor relapse free survival (GSE12276, *p* = 0.001; GSE1456-GPL97, *p* = 0.004) (Fig. 5a), and in one data set, low *GALNT9* expression was associated with poor relapse free survival, (GSE1379, *p* = 0.0029) (Fig. 5b). There was no evidence in any of the datasets analysed that low *BNC1* expression correlated with poor relapse free survival or any other clinical indicator.

## Discussion

Given the extremely poor clinical outcome following a diagnosis of BBM [9], it is imperative that the underlying molecular biology that drives tumour evolution to the colonization of the brain is elucidated.

To date, some progress has been made to identify prognostic markers for breast cancer metastasis by gene expression profiling [35]. However, prediction of site-specific metastasis remains poor [36].

The importance of gene dysregulation by promoter methylation as a mechanism of tumour evolution is now well established [37]. Indeed, genome wide methylation analysis of many hundreds of primary breast tumours has allowed the definition of specific sub-categories of breast tumours [12, 13], and our increasing understanding of the molecular basis of these subtypes has improved our ability to predict early metastatic recurrence [14, 38]. However, late recurrence, a common feature of BBM has proven difficult to predict [39].

We have carried out a broad candidate approach to identify genes that are dysregulated in BBM (Additional file 1: Figure S1). This analysis has identified three genes (*BNC1*, *CCDC8* and *GALNT9*) that are differentially methylated in primary breast tumours and BBM.

We predicted that our analysis of unrelated primary breast tumours and BBM would identify two different classes of genes that contribute to BBM, epigenetic silencing of BBM associated genes would either occur as (i) *early events* in tumour evolution that may be involved in processes such as local invasion and intravasion [40, 41] or these early events may be required for specific distant site metastasis but also contribute to primary tumour development or (ii) *late events* that play no significant role in the initial evolution of the primary tumour but contribute to the development of the secondary brain metastasis, perhaps by improving the capacity of these cells to survive in the foreign microenvironment of the brain.

The existence of early and late events had previously been proposed by Nguyen et al*.* [42], they classified deregulated genes as either involved in (i) metastasis initiation, detectable in the primary tumour, (ii) metastasis progression genes, important for survival in the circulation or required for extravasation, while occasionally present in the primary tumour, they may also occur once metastasising cells have left the primary site, or (iii) metastasis virulence genes that allow the cancer cells to survive in a foreign tissue environment. These are likely to occur as a consequence of the selection pressure provided by the novel environment the metastasised tumour cells find themselves in. Metastasis progression genes may have different functions in the primary tumour and distant metastasis, for example, *MMP-1* promotes vascular remodelling in primary breast tumours and also contributes to lung extravasation [43]. An example of a known metastasis virulence genes that does not contribute to primary tumour growth is interleukin-11, which promotes breast tumour metastasis to the bone but does not provide any advantage to the primary tumour [44].

Both early and late methylation events will appear similarly in our initial analysis; the genes will be frequently methylated in BBM and infrequently methylated in unrelated primary breast tumours, this is the case for *BNC1*, *CCDC8* and *GALNT9* (Fig. 1). However, a comparison of primary tumours and BBM from the same patient should reveal if specific gene methylation occurs early or late in the process of tumour evolution. Our analysis of such tumour pairs (Fig. 2) identified that *BNC1* and *GALNT9* are not frequently methylated in any breast tumours, even those that will eventually develop into brain metastases where these genes are methylated. Their methylation appears to be a late event in tumour evolution/metastasis. However, the *CCDC8* promoter was commonly methylated in primary breast tumours that eventually develop brain metastases and as such it can be categorised as an early event in tumour evolution/metastasis.

*GALNT9* encodes a member of the UDP-*N*-acetyl-α-d-galactosamine:polypeptide *N*-acetylgalactosaminyltransferase family of enzymes that catalyze the first step of O-glycosylation; GALNAC-T9. GLANT9 is expressed most abundantly in the brain and other CNS tissues. It is also expressed, at lower levels, in a number of other tissues including normal breast (GeneCards) [45].

The GALNAC-T proteins initiate mucin type O-linked glycosylation in the golgi apparatus by the covalent linkage of an α-*N*-acetylgalactosamine (GalNAc) to Ser and Thr residues [46]. O-Glycans play an important role in cell adhesion and cell-cell communication, and dysregulated glycosylation is a common characteristic of tumour cells [47]. Mucin 1 (MUC1), in particular, has been identified as a highly O-glycosylated transmembrane protein that is dysregulated at the expression and posttranslational level in multiple tumour types [47]. MUC1 is commonly overexpressed but under-glycosylated in primary breast tumours [48, 49], and the expression of under-glycosylated MUC1 is associated with high tumour grade, metastatic potential and invasiveness of breast tumours [50–52]. Loss of GALNT9 expression in neuroblastoma has been linked to a highly malignant phenotype and associated with poor overall and disease free survival [53]. GALNT9 is a member of a sub family (with GALNT8, 18 and 19) that differ significantly in sequence from other GALNAC-T members [54] and as such does not have catalytic activity towards classic MUC1 variants derivatives [55, 56]. This suggests that GALNT9 glycosylates a specific group of substrates indicating a subtle regulation of transmembrane protein function. Our findings of *GALNT9* promoter methylation, and associated loss of expression, in BBM, but not in primary breast tumours suggest that this change in transmembrane protein function may be a common occurrence in the later stages of breast tumour brain metastasis, and perhaps relates to cell-cell interaction that the tumour cells must undergo before acquiring a suitable niche to proliferate within the novel microenvironment of the brain.

This is the first time that *GLANT9* has been shown to be dysregulated in cancer by promoter methylation. However, conserved mutations have been identified in approximately 2 % of microsatellite instable colorectal cancers [57] and *GALNT9* is also mutated, infrequently (<1 %), in astrocytoma [58] and lung tumours [59, 60] and infrequently lost through CNV in breast tumours [12, 60].

Basonuclin 1 (BNC1) is a zinc finger transcription factor that interacts with the promoters of both RNA polymerases I and II [61]. BNC1 target genes have been implicated in a broad range of functions including chromatin structure, transcription/DNA-binding, adhesion, signal transduction and intracellular transport [61–63]. It is expressed in a broad range of tissue types (GeneCards) [45].

*BNC1* has previously been shown to be silenced by promoter methylation in lung [64], renal [31], pancreatic [65], prostate [66] and leukemic cancers [67]. In vitro assays have shown that loss of BNC1 expression is associated with an increased malignant phenotype [31]. Consistent with this study, analysis of HumanMethylation 27 and 450 K array data from *The Cancer Genome Atlas* indicates that *BNC1* Promoter methylation is an infrequent event in primary breast tumours [12]. However, frequent *BNC1* promoter methylation (>60 %) in a small cohort of breast tumours has previously been reported [64].

The expression of BNC1 is induced by transforming growth factor-β1 signalling and, in turn, it acts as a transcription factor for a number of modulators of epithelial dedifferentiation during EMT [68]. Moreover, loss of BNC1 expression results in a reduced EMT phenotype. These findings suggest that the expression of BNC1 would enhance the process of metastasis via EMT. Our findings are consistent with this; we find that *BNC1* is infrequently methylated in primary breast tumours (17 %) and frequently methylated and silenced in BBMs (73 %). Moreover, we have shown that *BNC1* promoter methylation is a late event in tumour evolution, only occurring in the brain metastasis of a BBM patient and not in the associated primary tumour. It is plausible that BNC1 expression is commonly required for EMT to occur during metastasis and, once these cells have metastasised to the brain, loss of *BNC1* expression contributes to mesenchymal to epithelial transition (MET).

An in vitro screen that consisted of multiple rounds of breast cancer cell line injection into nude mice and reculturing of the resulting brain metastases showed that *BNC1* was among a large number of genes overexpressed in mouse brain metastases [69]. This apparent difference to our findings may be as a consequence of the model used. Alternatively, it may represent important differences in the process of aggressive early metastasis (as cell line injection models represent) and slower metastatic evolution, where tumour cells proceed through a phase of latency or micrometastasis. Many of the brain metastases in our study were identified several years after initial breast cancer diagnosis (Paired primary and BBM samples were excised between 2 and 10 years apart).

*CCDC8* encodes a coiled-coil domain containing protein (CCDC8) that is one of three proteins that are mutated in patients with 3 M syndrome [70], an autosomal recessive disorder characterised by short stature, skeletal abnormalities, reduced male hormone and blood vessel bulges [71–73]. *CCDC8* is mutated in ~5 % of 3 M cases, the other genes, *CUL7* and *OBSL1* are mutated in ~65 % and ~30 % of cases, respectively [70, 74]. These three proteins form a complex (the 3 M complex) and loss of expression of any one protein disrupts microtubule dynamics resulting in dysregulated mitosis, cytokinesis, associated genomic instability and aneuploidy [75]. Moreover, it was shown that loss of any 3 M complex protein significantly altered the interphase microtubule network [75]. The core 3 M-protein complex interacts with CUL9, which has been proposed to mediate the functions of the 3 M complex via the ubiquitylation and degradation of survivin [76]. The 3 M-complex also interacts with the F box protein FBXW8, ROC1 and the tumour suppressor p53 [75] suggesting it may contribute to correct cellular physiology through multiple mechanisms.

Despite the broad range and very different known functions that these three proteins have it is interesting to see that, at the level of in vitro assays, reduced expression of any of them increases metastatic potential (Figs. 3, 4).

## Conclusions

Our findings indicate that epigenetic dysregulation of *GALNT9*, *CCDC8* or *BNC1* in breast tumours may contribute to metastasis to the brain and possibly other distant organs. *CCDC8* dysregulation occurs early during tumour evolution, in addition to being a potential therapeutic target this early inactivation has the potential to be utilised as a prognostic biomarker. Further analysis will be required including studies to determine if such epigenetic markers can be discerned via non-invasive means such as analysis of circulating tumour material in the patients blood. *GALNT9* and *BNC1* promoter methylation and associated silencing is common in BBM but does not occur frequently in the originating breast tumours suggesting that their dysregulation may not necessarily benefit the primary tumour but are required for successful colonization of the brain. Further studies will be required to determine if these changes are detectable in circulating tumour cells, micrometastases, or only in macroscopic brain metastases. Our current understanding of the cellular function of these genes is far from complete. However, what is known about all three suggests that their dysregulation may be more that just a marker for BBM. As such these genes may represent novel therapeutic targets.

## Additional files

Additional file 1: Figure S1. Graphical overview of methodologies used and results obtained in this study. (A) A literature review was carried out to identify genes that are methylated in lung, melanoma and renal cancer as these often metastasise to the brain rapidly. If these genes were not known to be frequently methylated in breast tumours (that metastasise to the brain with a longer lag period) they were considered as good candidates. (B) A literature review was carried out to identify genes down regulated in Epithelial to Mesenchmal Transition (EMT). (C) Analysis of genome-wide methylation data from *The Cancer Genome Atlas* identified 4 genes frequently methylated in Lung tumours and infrequently methylated in breast tumours with no evidence of distant metastasis. Genes from these candidate lists were screened for methylation in breast to brain metastases (BBM), those that were frequently methylated were then screened for methylation in non-metastatic primary breast tumours. Of the 82 genes analysed *BNC1*, *CCDC8* and *GALNT9* were frequently methylated in BBM and infrequently methylated in non-metastatic primary breast tumours, suggesting a role in the evolution of metastatic tumours. Additional file 2: Table S1. TCGA tumour barcodes: Unique barcode of Breast Invasive Carcinoma (BRCA) and Lung Adenocarcinoma (LUAD) tumours from *The Cancer Genome Atlas* (TCGA) downloaded for bioinformatic analysis to screen for candidate genes, which may contribute to breast to brain metastases (BBM). Additional file 3: Table S2. Molecular characteristics and other clinical information relating to primary breast tumours analyzed in this study. (A) Primary breast tumours that have metastasised to the brain. These primary tumours have associated metastases analysed in this study. (B) Primary breast tumours with no evidence of metastasis to the brain. These patients have no evidence of developing brain metastases, see Methods: Patients and samples for further details (N: Negative, P: Positive; 0: Negative, 1: Positive). Additional file 4: Table S3. Primers used in CoBRA and Reverse-Transcription (RT) PCR. CoBRA (Combined Bisulphite and Restriction Analysis) primers were designed to amplify promoter regions of 82 genes In addition, RT primers were designed to amplify transcripts of *BNC1*, *CCDC8* and *GALNT9* to investigate their expression in breast cancer cell lines and breast to brain metastases. F: Forward primer, IF: Internal Forward primer, IR: Internal Reverse primer, R: Reverse primer. Additional file 5: Figure S2. The promoter region/CpG islands of *BNC1, CCDC8* and *GALNT9.* The region amplified for CoBRA analysis is found between the Internal Forward primer and the Reverse primer. CpG dinucleotides are highlighted in bold. CpG dinucleotides analysed by cloning and sequencing of individual alleles are numbered. An arrow indicates the transcription start site. Additional file 6: Table S4. Genes analysed for their methylation status in breast to brain metastases (BBM) and their function. Methylation status of CpG island promoter region of 82 genes (4 genes from our bioinformatic screen and 78 genes from a broad literature review including genes down regulated in Epithelial- Mesenchymal Transition) was interrogated using Combined Bisulphite and Restriction Analysis (CoBRA) in BBM (n=15). 21 genes were frequently methylated in BBM (light grey background) of which, 3 genes (*CCDC8*, *BNC1* and *GALNT9*) (dark grey background) were infrequently methylated in an independent cohort of primary tumours (n=15). These three genes were further analysed in 20 more primary breast samples (n=30 in total) and 15 more BBM (n=30). Additional file 7: Figure S3. Methylation analysis of *BNC1*, *CCDC8* and *GALNT9* in Breast to brain metastases. Up to 31 brain metastases (BM) were analysed by CoBRA, small, digested PCR products in the Bstu1 cut (C) lane compared to the undigested (U) lane indicates promoter methylation in a sample (SAM DNA: genomic DNA treated with *S*-Adenosyl methionine and DNA methyltransferase as a positive control). Additional file 8: Figure S4. Methylation status of *GALNT9, CCDC8* and *BNC1* in metastatic brain tumours from primary breast tumours and a cohort of unrelated primary breast tumours. (A) *GALNT9* was frequently methylated in metastatic brain tumours (55 %) and (B) was NOT methylated in any of the primary tumours; (C) *CCDC8* was frequently methylated in metastatic brain tumours (73 %) and (D) infrequently methylated in primary breast tumours (40 %). (E) *BNC1* is frequently methylated in metastatic brain tumours 68 % and (F) infrequently methylated in a cohort of unrelated primary breast tumours (17 %). We have determined that a significant proportion of the promoters within the tumour sample are methylated if there are clearly observed digest products following restriction analysis BM: Brain metastases, BP: Primary breast tumours, U: Uncut/control sample, C: cut by restriction enzyme, *: methylated samples). Additional file 9: Figure S5. Bisulphite sequencing of individual alleles form tumours. Tumours were analysed by cloning and sequencing bisulphite-PCR products to determine the extent of methylation within the region analysed by CoBRA. 10 clones/alleles were sequenced for each tumour and the methylation index (MI) for each tumour determined. (A) Tumours that were determined to have significant *CCDC8* promoter methylation by CoBRA (BM11, BM15, BM12 and BM14) had methylation indices ranging from 66 %-90 %. The corresponding primary breast tumours for BM11 and BM15 were also analyses these both had correspondingly high MIs (82 % and 74 % respectively). Tumours that had no evidence of *CCDC8* promoter region methylation by CoBRA analysis (BM16, BM23) had low MIs (8 % and 6 % respectively). (B) Tumours that were determined to have significant *BNC1* promoter methylation by CoBRA (BM13, BM14, BM15 and BM27) had methylation indices ranging from 60 %-86 %. Tumours that had no evidence of *BNC1* promoter region methylation by CoBRA analysis (BM11, BM23) had low MIs (0 % and 36 % respectively). (C) Tumours that were determined to have significant *GALNT9* promoter methylation by CoBRA (BM12, BM20, BM27 and BM28) had methylation indices ranging from 78 %-91 %. Tumour BM23 that had no evidence of *GALNT9* promoter region methylation by CoBRA analysis had a low MI (25 %). Each circle represents a CpG island, those shaded black are methylated. MI is defended as the total number of methylated CpG dinucleotides given as a percentage of all CpGs analysed. Additional file 10: Figure S6. Expression levels of *BNC1. CCDC8* and *GALNT9* in all tumours analysed. The expression level of each gene was quantified in relation to the expression of β-actin. Below each bar is the methylation status of each CpG island as determined by CoBRA and sequencing of individual alleles (MI) (BM: Brain Metastasis, MI: Methylation index, M: Methylated, U: Unmethylated, -: analysis was not done). Additional file 11: Figure S7. Global demethylation resulted in the re-expression of *GALNT9*, *CCDC8* and *BNC1* in breast cancer cell lines. Reverse transcription PCR (RT-PCR) showed that treatment of breast cancer cell lines with 5- 2-deoxycytidine (5-AZA-dC), an inhibitor of DNA methyltransferase enzymes, resulted in re-expression of (A) *GALNT9,* (B) *CCDC8* or (C) *BNC1* in the breast cancer cell line ZR75. For comparison, endogenous expression is shown in (A) MDA-MD231, (B) T47D and (C) MCF7, these, expressing, cell lines were used in our *in vitro* knock down experiments. Additional file 12: Figure S8. Knockdown of *GALNT9*, *CCDC8,* and *BNC1* in breast cancer cell lines is confirmed by Reverse transcription (RT) PCR and western blot. (A) RT-PCR of *GALNT9*, *CCDC8,* and *BNC1* transcripts in breast cancer cell lines (MDA-MD231, T47D and MCF7 respectively) following siRNA knockdown compared to transfection with a control siRNA and (B) western blot of GALNT9, CCDC8*,* and BNC1 proteins to confirm their knockdown in each respective cell line. 70 μg of protein was loaded in each lane. Equal loading was confirmed by staining total protein with India ink.

## Abbreviations

5-AZA-dC: 5-AZA-2′-deoxycytidine
BBM: Breast to brain metastasis
BM: Brain metastasis
BN: Normal tissue adjacent to breast tumour
BP: Breast primary tumour
CoBRA: Combined bisulfite and restriction analysis
EMT: Epithelial to mesenchymal Transition
ER: Estrogen receptor
FFPE: Formalin fixed paraffin embedded
HER2: Human epidermal growth factor receptor 2 (ERBB2)
MET: Mesenchymal to epithelial transition
MI: Methylation index
PR: Progesterone receptor
RCC: Renal cell carcinoma
SAM: S-adenosyl methionine
TCGA: The Cancer Genome Atlas
