# Supplementary material for: The GALNT9, BNC1 and CCDC8 genes are frequently epigenetically dysregulated in breast tumours that metastasise to the brain
Source: Clin Epigenetics. 2015 May 27;7(1):57. doi: 10.1186/s13148-015-0089-x (PMC4457099; doi:10.1186/s13148-015-0089-x)

Supplementary Figure 1: Graphical overview of methodologies used and results obtained in this study

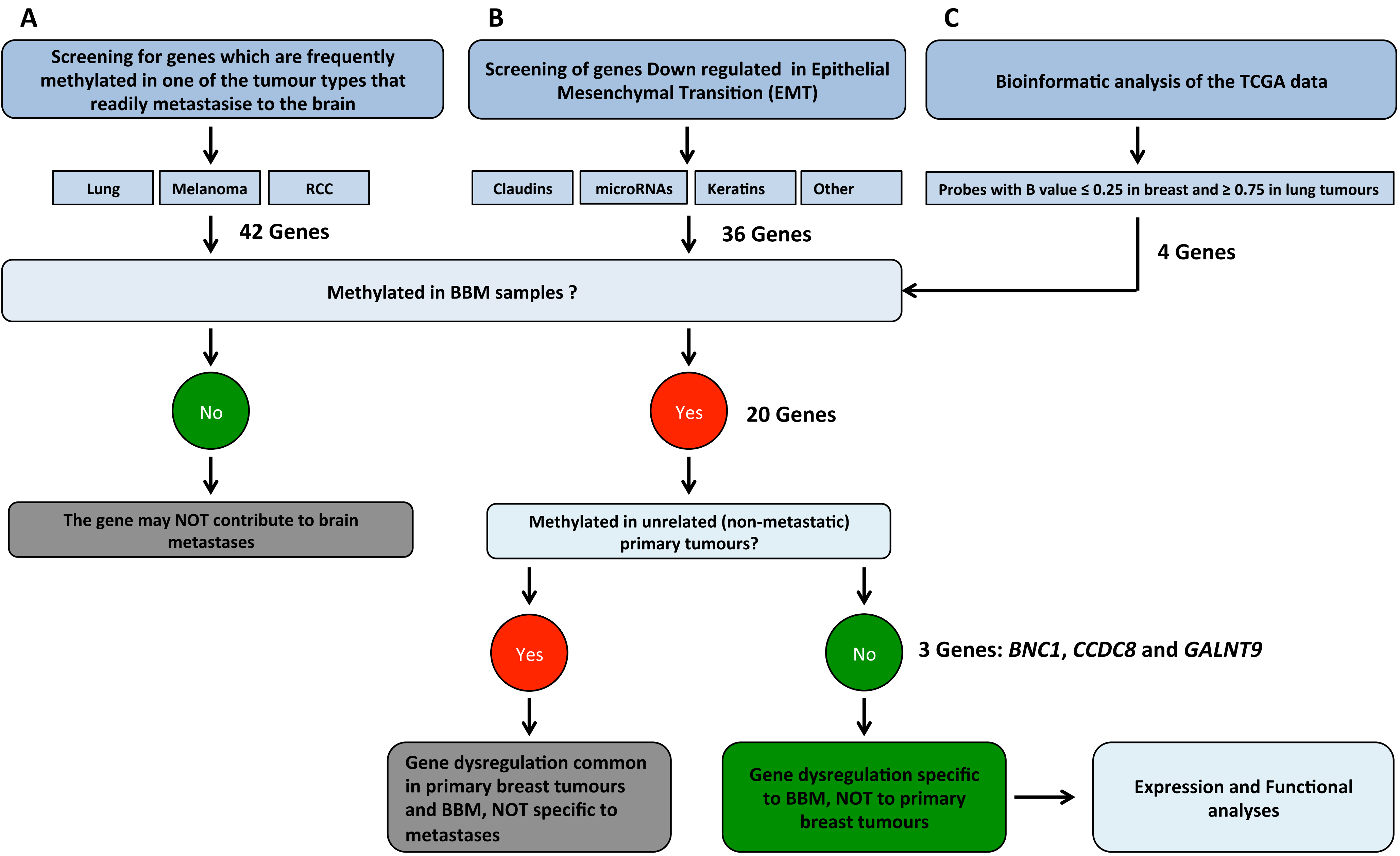

Supplement: Additional file 1: Figure S1. — Graphical overview of methodologies used and results obtained in this study. (A) A literature review was carried out to identify genes that are methylated in lung, melanoma and renal cancer as these often metastasise to the brain rapidly. If these genes were not known to be frequently methylated in breast tumours (that metastasise to the brain with a longer lag period) they were considered as good candidates. (B) A literature review was carried out to identify genes down regulated in Epithelial to Mesenchmal Transition (EMT). (C) Analysis of genome-wide methylation data from The Cancer Genome Atlas identified 4 genes frequently methylated in Lung tumours and infrequently methylated in breast tumours with no evidence of distant metastasis. Genes from these candidate lists were screened for methylation in breast to brain metastases (BBM), those that were frequently methylated were then screened for methylation in non-metastatic primary breast tumours. Of the 82 genes analysed BNC1, CCDC8 and GALNT9 were frequently methylated in BBM and infrequently methylated in non-metastatic primary breast tumours, suggesting a role in the evolution of metastatic tumours. [file 13148_2015_89_MOESM1_ESM.pdf]
