# Supplementary material for: The GALNT9, BNC1 and CCDC8 genes are frequently epigenetically dysregulated in breast tumours that metastasise to the brain
Source: Clin Epigenetics. 2015 May 27;7(1):57. doi: 10.1186/s13148-015-0089-x (PMC4457099; doi:10.1186/s13148-015-0089-x)
Supplement: Additional file 5: Figure S2. — The promoter region/CpG islands of BNC1, CCDC8 and GALNT9. The region amplified for CoBRA analysis is found between the Internal Forward primer and the Reverse primer. CpG dinucleotides are highlighted in bold. CpG dinucleotides analysed by cloning and sequencing of individual alleles are numbered. An arrow indicates the transcription start site. [file 13148_2015_89_MOESM5_ESM.pdf]

BNC1 Region of analysis

Forward Primer

CCTGAGAAGAGCGCCAGAGAACTTCAGAGCGTTTCGCCCTTCCCCGGAGAGGCAAACAC

Internal Forward Primer
1

CGACACGTCTGTGTCTTTTACCAACAAGTGCCTTCAAGCCCGGCGGGGCAGACACCTCC

2 3 4 5 6 7 8 9 10 11 12

GCGCCGGCCGCCGGCGAGGTCTCCGCGGTCTGCGGGGGCCACGGCCTCGCCTCAGCTGCG

13 14 15 16 17 18 19 20

CTGATTTAGGGCGTTATCCGGTCCCGGGGCGGAGGCGGCCTCCCGGGCGGCGAAGCAGG

21 22 23 24 25 26 27 28 29

GCCCGCGGCGTGGGGCGACCGCGCGGTGGGCGGAGGGCAGGGGAGGGGCGGAGAGGCG

TCCCCGGGGCGCAGGGGGCGGGCGTGCGGGCACACGCGGTGCGCGGCGGGGGCGGCCATC

Reverse Primer

GTGCTGCGCAGCCTGGGCGCTTGGGGAGCCGCCCACTTCGCCGGGTCGCGCCCCGACGGC

CCDC8 Region of analysis

Forward Primer

GCTGACGTGGGCCACTGCCGCTTCGCTGGGAAGCAATGGCCCAGCTAGGCCCGGGGCGC

Internal Forward Primer

GGCCACACCCCTGTGGGGAGGGGAAGGAGGCCGCCCCGAAGGGAGTGGACAGCCCCCCT

1

GTCAGTCTTCCAGAGTCTGGGAGTGTAAGATGAGACGGGGGAAGGTGGCCTCATTCTGG

2 3 4 5 6 7 8 9 10

CGGAGGGCGAGGAGGAACTTCCTGCCCGCGCGCTCCACGGTGCAGAGCTCTAAGCGCGCG

11 12 13 14

GGCTGGCAGGCTGCGGCGCGTCAAGGTCAGCCTGGAGCTGGGTGGCGGCCTGCCTGGGGG

15 16 17 18

CGGGGGACCCTACTGGAGGCCCGGGCTGGGGCCTCCCAGCGCCTCGGCCATATTGAATAG

19 20 21 22 23

CTTCGACTGGACCGTCTTTGTCTGCGAAGTCCTGTCCCAAGTTCCAGCCGCGTCCCTGGG

24 25 26 27

GCCTGGGGCAGGAAGAGTCGCTGGCAGCCCGCGCGCCCCAACTTGGAGCTGGGACACCAC

Reverse Primer

GTTTCCAGCTTGGAGTGGCCTTGAGCCTTGGACTGACCTCGCCCCGGCTCACGTAGG

GALNT9 Region of analysis

Forward Primer

CGCGGTTGCAGATGAGGTGAGGTGAGGCCGCGTCACTCTGCACCGGCGCGGTGGCTGCGG

Internal Forward Primer

GGCGGGCAGGACAGGAGCCGGCACAGACACCGAGCGCCGCCCGCCCGCGCCTTCCCCGCC

1 2 3 4

GCCCCCCGGCGCCCCCGGCCCCCCTCACCGCTCCCCGGGGCGGGGCCGCGCCCTCTGAGC

5 6 7 8 9 10 11

GGGGATGCCGGCCGCGCCCCGCGACCCCAGCCCCGGGCAGCCCTCTGCGCTCTGGGGA

12 13 14 15 16 17 18 19

CCCCCGGCGGCCGTGGCCCGGCGCGCTGAGCTGGTGCTGAAGGACAGCTCCGGCCGAGC

20 21 22 23

CCCGCAGCCCCCGCAGCCCCGGGCGGCTCATGGTCCCCGAAGCCGAAGCTGAAGCCCAGG

CCCGGGCGGGGATGCTGGGGATGCCCCGCGGGTGAGGCCCCCGCTGCAGCCGTGTTCATG

Reverse Primer

GCGGTGGCCAGGAAGATCCGAACTTTGCTGACGGTGAACATCCTGGTGTTCGTGGGCATC
