# Supplementary material for: The GALNT9, BNC1 and CCDC8 genes are frequently epigenetically dysregulated in breast tumours that metastasise to the brain
Source: Clin Epigenetics. 2015 May 27;7(1):57. doi: 10.1186/s13148-015-0089-x (PMC4457099; doi:10.1186/s13148-015-0089-x)
Supplement: Additional file 6: Table S4. — Genes analysed for their methylation status in breast to brain metastases (BBM) and their function. Methylation status of CpG island promoter region of 82 genes (4 genes from our bioinformatic screen and 78 genes from a broad literature review including genes down regulated in Epithelial- Mesenchymal Transition) was interrogated using Combined Bisulphite and Restriction Analysis (CoBRA) in BBM (n=15). 21 genes were frequently methylated in BBM (light grey background) of which, 3 genes (CCDC8, BNC1 and GALNT9) (dark grey background) were infrequently methylated in an independent cohort of primary tumours (n=15). These three genes were further analysed in 20 more primary breast samples (n=30 in total) and 15 more BBM (n=30). [file 13148_2015_89_MOESM6_ESM.docx]

**Supplementary table 4**

| **Gene symbol** | **Accession** | **Gene name** | **% of tumours methylated** | **Function** |
| --- | --- | --- | --- | --- |
| *CLDN18* | NM_016369.3 | *Claudin 18* | 100 | Intercellular adhesion molecule responsible for tight junction strand formation [1] |
| *KRT85* | NM_002283.3 | *Keratin 85* | 100 | Component of intermediate filament in epithelial cells contributing to cell-cell adhesion [2-4] |
| *MIR127* | NR_029696.1 | *microRNA 127* | 100 | Regulator of cell proliferation and senescence [5] |
| *MIR433* | NR_029966.1 | *microRNA 433* | 100 | Deregulated in gastric cancer, regulator of cell migration and drug response [6, 7] |
| *HOXD3* | NM_006898.4 | *HomeoboxD3* | 100 | Proangiogenic transcription factor [8] |
| *MIR23B* | NR_029664.1 | *microRNA 23b* | 92 | Involved in cytoskeleton modelling, motility and metastasis [9-12] |
| *CCDC8* | NM_032040.4 | *Coil coiled domain containing 8* | 87 | Mutated in patients with 3M syndrome [13]. Loss is associated with genomic instability and aneuploidy [14]. |
| *KRT83* | NM_002282.3 | *Keratin 83* | 84 | Component of intermediate filament, contributes to cell to cell adhesion [2, 4] |
| *HOXB13* | NM_006361.5 | *Homeobox B13* | 80 | TSG for prostate cancer, inhibits androgen mediated signalling [15] |
| *ABCB1* | NM_000927.4 | *ATP-binding cassette sub-family B member 1* | 80 | Controls efflux of substances across plasma membranes, associated with multidrug resistance [16] |
| *PENK* | NM_006211.3 | *Proenkephalin* | 80 | Promotes RNA splicing in osteoblasts and neural cells, plays role in bone development [17] |
| *MST1R* | NM_002447.2 | *macrophage stimulating 1 receptor* | 78 | Involved in intracellular signalling cascades leading to cellular growth, motility and invasion [18] |
| *BNC1* | NM_001717.3 | *Basonuclin 1* | 73 | Zink finger transcription factor, regulator of EMT [19] |
| *PCDH8* | NM_002590.3 | *Procadhern 8* | 73 | Helps in cell to cell adhesion [20] |
| *STAT3* | NM_139276.2 | *Signal transducer and activator of transcription 3* | 67 | Involved in embryonic stem cell regulation, somatic cell growth [21-23] |
| *BVES* | NM_007073.4 | *Blood vessel epicardial substance* | 64 | Involved in inter-cellular interaction and cell adhesion. [24] |
| *TNFRSF10D* | NM_003840.4 | *Tumour Necrosis Factor receptor superfamily 10 D* | 60 | Member of TNF (Tumour Necrosis Factor) receptor superfamily, promotes apoptosis in cancer cells [25] |
| *CLDN6* | NM_021195.4 | *Claudin 6* | 55 | Intercellular adhesion molecules responsible for tight junction strand formation, its epigenetic silencing is associated with migration and invasiveness of breast cancer [1, 26] |
| *HOXD10* | NM_002148.3 | *Homeobox D10* | 55 | Maintain epithelial cell plasticity and contributes to stability of extracellular matrix [27] |
| GALNT9 | NM_001122636.1 | *N-acetylgalactosaminyltransferase 9* | 55 | Catalyzes O-glycosylation [28, 29] |
| *WIF1* | NM_007191.4 | *Wnt inhibitory factor-1 gene* | 53 | Inhibitor of Wnt-signalling [30, 31] |
| *CLDN5* | NM_001130861.1 | *Claudin 5* | 47 | Intercellular adhesion molecule responsible for tight junction strand formation, involved in breast cancer metastasis, its loss affects blood brain barrier selectivity [1, 32, 33] |
| *SFRP2* | NM_003013.2 | *Secreted frizzled-related protein 2* | 47 | Regulator of canonical Wnt pathway [34] |
| *KRT72* | NM_080747.2 | *Keratin 72* | 42 | Component of intermediate filament, contributes to cell- cell adhesion [2, 4] |
| *MIR124-1* | NR_029668.1 | *microRNA 124-1* | *36* | miRNA with tumour suppressor activity, epigenetically deregulated in various cancers [35-37] |
| *MIR34b* | NR_029839.1 | *microRNA 34b* | 30 | Tumour suppressor miRNA, associated with p53 regulation, cancer and apoptosis [38] |
| *CLDN1* | NM_021101.4 | *Claudin 1* | 20 | Intercellular adhesion molecule responsible for tight junction strand formation [1, 39-41] |
| *BOLL* | NM_197970.2 | *boule-like* | 13 | Associated with spermatogenesis [42] |
| *COL14A1* | NM_021110.2 | *Collagen, type XIV, alpha-1* | 13 | Interacts with extracellular matrix components, cell adhesion [43] |
| *DAPK* | NM_004938.3 | *Death Associated protein kinase* | 13 | Serine/threonine kinase involved in the apoptotic system [44] [45] |
| *TNFRSF10C* | NM_003841.3 | *Tumour Necrosis Factor receptor superfamily 10C* | 13 | Cell surface protein, modulates multiple biological networks [46] |
| *DGKI* | NM_004717.2 | *Diacylglycerol kinase* | 13 | Activator of signalling proteins [47] |
| *MIR34a* | NR_029610.1 | *microRNA 34* | 10 | Transcriptional target of p53 associated with apoptosis and metastasis [38, 48-50] |
| *GREM1* | NM_013372.6 | *Gremlin1* | 7 | Tissue re-modelling and angiogenesis [51] |
| *CDKN1A* | NM_000389.4 | *Cyclin-Dependent Kinase Inhibitor 1A* | 7 | Cell cycle regulator [52] |
| *AK5* | NM_174858.2 | *Adenylate kinase* | 0 | Phosphoryl exchange [53] |
| *ALDH1A3* | NM_000693.2 | *Aldehyde dehydrogenase* | 0 | Retinal oxidation [54] |
| *ANK3* | NM_020987.3 | *Ankyrin-3/G* | 0 | Regulates voltage gated sodium channels [55] |
| *ATM* | NM_000051.3 | *Ataxia telangiectasia mutated* | 0 | Key regulator in multiple signalling cascades [56] |
| *CD44* | NM_000610.3 | *CD44 molecule* | 0 | Main receptor for extracellular matrix component Hyaluronan (HA) [57] |
| *CLDN11* | NM_005602.5 | *Claudin 11* | 0 | Intercellular adhesion molecule re [1] |
| *CLDN23* | NM_194284.2 | *Claudin 23* | 0 | Intercellular adhesion molecule [1, 58] |
| *CLDN3* | NM_001306.3 | *Claudin 3* | 0 | Intercellular adhesion molecule [1, 58] |
| *CLDN4* | NM_001305.4 | *Claudin 4* | 0 | Intercellular adhesion molecule [1, 58] |
| *CLDN7* | NM_001185022.1 | *Claudin 7* | 0 | Intercellular adhesion molecule [1, 59] |
| *CLDN9* | NM_020982.3 | *Claudin 9* | 0 | Intercellular adhesion molecule [1] |
| *CMTM8* | NM_178868.3 | *CKLF-like MARVEL transmembrane domain containing 8* | 0 | Negative regulator of EGM induced signalling, induces apoptosis [60] |
| *CSNK1A1* | NM_001025105.2 | *Casein kinase 1, alpha 1* | 0 | Serine/Threonine kinase plays role in cellular senescence [61] |
| *DLC1* | NM_182643 | *Deleted in Liver Cancer* | 0 | GTPase activating protein plays roles in signalling pathways [62] |
| *DSP* | NM_004415.2 | *Desmoplakin* | 0 | Inhibits Wnt/Beta catenin pathway [63-66] |
| *FBN2* | NM_001999.3 | *fibrillin 2* | 0 | Associated with formation of microfibrils and elastic fibrillogenesis [67] |
| *FBXL14* | NM_152441.2 | *F-box and leucine-rich repeat protein 14* | 0 | Hypoxia induced ubiquitin ligase, targets  *SNAIL1* [68, 69] |
| *GATA5* | NM_080473.4 | *GATA binding protein 5* | 0 | Transcription factor [70] |
| *HK2* | NM_000189.4 | *Hexokinase* | 0. | First rate limiting enzyme of glycolysis, involved in pancreatic carcinogenesis [71] [72] |
| *ICAM5* | NM_003259.3 | *intercellular adhesion molecule 5* | 0 | Cellular adhesion Involved in dendritic outgrowth [73] |
| *IGFBP3* | NM_000598.4 | *Insulin-like growth factor-binding protein 3* | 0 | Regulator of cell growth and apoptosis [74] |
| *KLHL35* | NM_001039548.2 | *Kelch –like 35* | 0 | Associated with embryogenesis and cancers [75] |
| *KRT18* | NM_000224.2 | *Keratin 18* | 0 | Component of intermediate filament in epithelial cells contributing to cell to cell adhesions, involved in apoptosis and is associated with invasiveness of breast cancer  [2, 4, 76] [77] |
| *KRT19* | NM_002276.4 | *Keratin 19* | 0 | Component of intermediate filament in epithelial cells contributing to cell to cell adhesion [2, 4] |
| *KRT28* | NM_181535.3 | *Keratin 28* | 0 | Component of intermediate filament in epithelial cells contributing to cell to cell adhesions [2, 4] |
| *KRT7* | NM_005556.3 | *Keratin 7* | 0 | Component of intermediate filament in epithelial cells contributing to cell to cell adhesions [2, 4] |
| *KRT81* | NM_002281.3 | *Keratin 81* | 0 | Component of intermediate filament in epithelial cells contributing to cell to cell adhesions [2, 4] |
| *KRT86* | NM_002284.3 | *Keratin 86* | 0 | Component of intermediate filament in epithelial cells contributing to cell to cell adhesions [2, 4] |
| *MMP2* | NM_004530.4 | *Matrix metallopeptidase 2* | 0 | Extracellular enzyme ,promotes cell migration and invasion [78] |
| *NRCAM* | NM_001037132.2 | *Neuronal cell adhesion molecule* | 0 | Neural-glial cell adhesion molecule, involved in proliferation and signalling pathways [79] |
| *OCLN* | NM_002538.3 | *Occludin* | 0 | Tight junction associated integral protein, [80] |
| *PBRM1* | NM_018313.4 | *polybromo 1* | 0 | Regulates embryonic development associated with renal cell carcinoma [81] |
| *PNN* | NM_002687.3 | *pinin, desmosome associated protein* | 0 | Associated with linking intermediate filaments to desmosome in epithelial cells [82] |
| *PTEN* | NM_000314 | *Phosphatase and tensin homolog* | 0 | Modulates cell signalling, growth, migration and apoptosis, regulates PI3K pathway [83, 84] |
| *PYCARD* | NM_013258.4 | *Apoptosis-associated speck-like protein containing a CARD* | 0 | Pro-apoptosis regulator [85] |
| *QPCT* | NM_012413.3 | *Glutaminyl-peptide cyclotransferase* | 0 | CCL2 signalling [86] |
| *RBP1* | NM_002899.3 | *retinol binding protein 1* | 0 | Retinol transport and metabolism, associated with ovarian cancer [87] |
| *SDHD* | NM_001276503.1 | *Succinate dehydrogenase complex, subunit D* | 0 | Respiratory chain protein [88] |
| *SULF2* | NM_018837.3 | *sulfatase 2* | 0 | Modulates signalling proteins and inhibit tumour growth [89] |
| TFAP2A | NM_003220.2 | *Transcription factor AP-2 alpha* | 0 | Required for neural crest induction [90] |
| *TJP1* | NM_003257.3 | *tight junction protein 1* | 0 | Tight junction organization and assembly [91] |
| *TMEFF2* | NM_016192.2 | *Transmembrane protein with EGF-like and two follistatin-like domains 2* | 0 | Transmembrane protein [92] |
| *TSC1* | NM_000368.4 | *Tuberous Sclerosis 1* | 0 | Regulator of cellular proliferation [93] |
| *TSPAN13* | NM_014399.3 | *Tetraspanin 13* | 0 | Transmembrane protein, inhibits cellular growth and invasion [94] |
| TSPAN4 | NM_001025237.1 | *Tetraspanin4* | 0 | Cellular growth, adhesion and differentiation [95] |
| *UCHL1* | NM_004181.4 | *Ubiquitin carboxyl-terminal esterase L1* | 0 | ubiquitinating enzyme, associated with early onset of progressive neurodegeneration [96] |
| ZNF808 | NM_001039886.3 | *Zinc finger protein 808* | 0 | Zinc finger protein, may be involved in transcriptional regulation [97] |

1. M, T.S.a.F., *The Structure and Function of Claudins, Cell Adhesion Molecules at Tight Junctions.* Ann N Y Acad Sci, 2000. **915**: p. 129-135.

2. Bragulla, H.H. and D.G. Homberger, *Structure and functions of keratin proteins in simple, stratified, keratinized and cornified epithelia.* J Anat, 2009. **214**(4): p. 516-59.

3. Shimomura, Y., et al., *Mutations in the keratin 85 (KRT85/hHb5) gene underlie pure hair and nail ectodermal dysplasia.* J Invest Dermatol, 2010. **130**(3): p. 892-5.

4. Magin, T.M., P. Vijayaraj, and R.E. Leube, *Structural and regulatory functions of keratins.* Exp Cell Res, 2007. **313**(10): p. 2021-32.

5. Chen, J., et al., *miR-127 regulates cell proliferation and senescence by targeting BCL6.* PLoS One, 2013. **8**(11): p. e80266.

6. Luo, H., et al., *Down-regulated miR-9 and miR-433 in human gastric carcinoma.* J Exp Clin Cancer Res, 2009. **28**: p. 82.

7. Symmans, W.F., *Tissue banking.* Breast Cancer Research, 2010. **12**(Suppl 1): p. L1.

8. Chen, Y., et al., *Retroviral delivery of homeobox D3 gene induces cerebral angiogenesis in mice.* Journal of cerebral blood flow and metabolism : official journal of the International Society of Cerebral Blood Flow and Metabolism, 2004. **24**(11): p. 1280-7.

9. Pellegrino, L., et al., *miR-23b regulates cytoskeletal remodeling, motility and metastasis by directly targeting multiple transcripts.* Nucleic Acids Res, 2013. **41**(10): p. 5400-12.

10. Jin, L., et al., *Prooncogenic factors miR-23b and miR-27b are regulated by Her2/Neu, EGF, and TNF-alpha in breast cancer.* Cancer Res, 2013. **73**(9): p. 2884-96.

11. Majid, S., et al., *miR-23b represses proto-oncogene Src kinase and functions as methylation-silenced tumor suppressor with diagnostic and prognostic significance in prostate cancer.* Cancer Res, 2012. **72**(24): p. 6435-46.

12. Zaman, M.S., et al., *Inhibition of PTEN gene expression by oncogenic miR-23b-3p in renal cancer.* PLoS One, 2012. **7**(11): p. e50203.

13. Hanson, D., et al., *Exome sequencing identifies CCDC8 mutations in 3-M syndrome, suggesting that CCDC8 contributes in a pathway with CUL7 and OBSL1 to control human growth.* American journal of human genetics, 2011. **89**(1): p. 148-53.

14. Yan, J., et al., *The 3M complex maintains microtubule and genome integrity.* Mol Cell, 2014. **54**(5): p. 791-804.

15. Kim, Y.R., et al., *HOXB13 promotes androgen independent growth of LNCaP prostate cancer cells by the activation of E2F signaling.* Molecular cancer, 2010. **9**: p. 124.

16. Muggerud, A.A., et al., *Frequent aberrant DNA methylation of ABCB1, FOXC1, PPP2R2B and PTEN in ductal carcinoma in situ and early invasive breast cancer.* Breast cancer research : BCR, 2010. **12**(1): p. R3.

17. H Rosen, A.K., R D Polakiewicz, S Benzakine, and Z Bar-Shavit, *Developmental regulation of proenkephalin gene expression in osteoblasts.* Molecular Endocrinology, 2013. **9**(11): p. 1621-1631.

18. Wagh, P.K., B.E. Peace, and S.E. Waltz, *Met‐Related Receptor Tyrosine Kinase Ron in Tumor Growth and Metastasis.* 2008. **100**: p. 1-33.

19. Feuerborn, A., et al., *Basonuclin-1 modulates epithelial plasticity and TGF-beta1-induced loss of epithelial cell integrity.* Oncogene, 2014.

20. Sabine Strehl, K.G., Qiu, Mei Liu, Heather Glatt, Marc, Lalande, *Characterization of Two Novel Protocadherins (PCDH8 and PCDH9) Localized on Human Chromosome 13 and Mouse Chromosome 14.* GENOMICS, 1998. **53**: p. 81-89.

21. Hitoshi Niwa, 2 Tom Burdon,1 Ian Chambers, and Austin Smith, *Self-renewal of pluripotent embryonic stem cells is mediated via activation of STAT3.* GENES & DEVELOPMENT, 1998. **12**: p. 2048-2060.

22. KIYOSHI TAKEDA, K.N., WEI SHI, TAKASHI TANAKA, MAKOTO MATSUMOTO, NOBUAKI YOSHIDA, TADAMITSU KISHIMOTO†, AND SHIZUO AKIRA, *Targeted disruption of the mouse Stat3 gene leads to early embryonic lethality.* proc. Natl. Acad. Sci; Developmental biology, 1997. **94**: p. 3801-3804.

23. Akira, S., *Roles of STAT3 de®ned by tissue-speci®c gene targeting.* Oncogene. **19**: p. 2607-2611.

24. Osler, M.E., T.K. Smith, and D.M. Bader, *Bves, a member of the Popeye domain-containing gene family.* Dev Dyn, 2006. **235**(3): p. 586-93.

25. Hill, V.K., et al., *Genome-wide DNA methylation profiling of CpG islands in breast cancer identifies novel genes associated with tumorigenicity.* Cancer Res, 2011. **71**(8): p. 2988-99.

26. Osanai, M., et al., *Epigenetic silencing of claudin-6 promotes anchorage-independent growth of breast carcinoma cells.* Cancer Sci, 2007. **98**(10): p. 1557-62.

27. Carrio, M., et al., *Homeobox D10 induces phenotypic reversion of breast tumor cells in a three-dimensional culture model.* Cancer Res, 2005. **65**(16): p. 7177-85.

28. Shinya Toba , M.T., Morichika Konishi Tadahisa Mikami et al, *Brain-speci¢c expression of a novel human UDP-GalNAc:polypeptide*

*N-acetylgalactosaminyltransferase (GalNAc-T9).* Biochimica et Biophysica Acta, 2000. **1493**: p. 264-268.

29. Berois, N., et al., *GALNT9 gene expression is a prognostic marker in neuroblastoma patients.* Clin Chem, 2013. **59**(1): p. 225-33.

30. Ai, L., et al., *Inactivation of Wnt inhibitory factor-1 (WIF1) expression by epigenetic silencing is a common event in breast cancer.* Carcinogenesis, 2006. **27**(7): p. 1341-8.

31. Veeck, J., et al., *Prognostic relevance of Wnt-inhibitory factor-1 (WIF1) and Dickkopf-3 (DKK3) promoter methylation in human breast cancer.* BMC cancer, 2009. **9**: p. 217.

32. Escudero-Esparza, A., W.G. Jiang, and T.A. Martin, *Claudin-5 is involved in breast cancer cell motility through the N-WASP and ROCK signalling pathways.* J Exp Clin Cancer Res, 2012. **31**: p. 43.

33. Nitta, T., et al., *Size-selective loosening of the blood-brain barrier in claudin-5-deficient mice.* J Cell Biol, 2003. **161**(3): p. 653-60.

34. Veeck, J., et al., *Promoter hypermethylation of the SFRP2 gene is a high-frequent alteration and tumor-specific epigenetic marker in human breast cancer.* Molecular cancer, 2008. **7**: p. 83.

35. Wilting, S.M., et al., *Methylation-mediated silencing and tumour suppressive function of hsa-miR-124 in cervical cancer.* Mol Cancer, 2010. **9**: p. 167.

36. Shi, X.B., et al., *Tumor suppressive miR-124 targets androgen receptor and inhibits proliferation of prostate cancer cells.* Oncogene, 2013. **32**(35): p. 4130-8.

37. Wong, K.Y., et al., *Epigenetic inactivation of the miR-124-1 in haematological malignancies.* PLoS One, 2011. **6**(4): p. e19027.

38. Hermeking, H., *The miR-34 family in cancer and apoptosis.* Cell Death Differ, 2010. **17**(2): p. 193-9.

39. Myal, Y., E. Leygue, and A.A. Blanchard, *Claudin 1 in breast tumorigenesis: revelation of a possible novel "claudin high" subset of breast cancers.* J Biomed Biotechnol, 2010. **2010**: p. 956897.

40. Weber, F.K.m.K.W.M.K.K.S.B.H.F., *Genomic organization of claudin-1 and its assessment in hereditary and sporadic breast cancer.* Human Genetics, 2000. **107**(3): p. 249-256.

41. Fritzsche, F.R., et al., *Claudin-1 protein expression is a prognostic marker of patient survival in renal cell carcinomas.* Clin Cancer Res, 2008. **14**(21): p. 7035-42.

42. Westerveld, G.H., et al., *Mutations in the human BOULE gene are not a major cause of impaired spermatogenesis.* Fertil Steril, 2005. **83**(2): p. 513-5.

43. Michael Bauer, W.D., Tobias Ehnis, Detlef Schuppan *Complete primary structure of human collagen type XIV ž Undulin/.* Biochimica et Biophysica Acta, 1997(1354): p. 183-188.

44. Pulling, L.C., et al., *Dual promoter regulation of death-associated protein kinase gene leads to differentially silenced transcripts by methylation in cancer.* Carcinogenesis, 2009. **30**(12): p. 2023-30.

45. Shinichi Toyooka, K.O.T., Kuniharu Miyajima, et al., *Epigenetic Down-Regulation of Death-associated Protein Kinase in Lung Cancers.* Clinical Cancer Research, 2003. **9**: p. 3034-3041.

46. Mariapia A. Degli-Esposti, P.J.S., Henning Walczak, Jennifer Waugh, Chang-Pin Huang, Robert F. DuBose, and a.C.A.S. Raymond G. Goodwin, *Cloning and characterization of TRAIL-R3, a novel member of the emerging TRAIL receptor family.* J Exp Med, 1997. **186**(7): p. 1165-1170.

47. Regier, D.S., et al., *Diacylglycerol kinase iota regulates Ras guanyl-releasing protein 3 and inhibits Rap1 signaling.* Proc Natl Acad Sci U S A, 2005. **102**(21): p. 7595-600.

48. Rokhlin OW, S.V., Taghiyev AF, Bumcrot D, Glover RA, Cohen MB, *MicroRNA-34 mediates AR-dependent p53-induced apoptosis in prostate cancer*

*.* Cancer Biol Ther, 2008. **7**(8): p. 1288-1296.

49. Roy S, L.E., Majumdar APN, Sarkar FH, *Expression of miR-34 is lost in colon cancer which can be re-expressed by a novel agent CDF.* Journal of Hematology and Oncology, 2012. **5**: p. 58-63.

50. Yamakuchi, M., M. Ferlito, and C.J. Lowenstein, *miR-34a repression of SIRT1 regulates apoptosis.* Proc Natl Acad Sci U S A, 2008. **105**(36): p. 13421-6.

51. Mulvihill, M.S., et al., *Gremlin is overexpressed in lung adenocarcinoma and increases cell growth and proliferation in normal lung cells.* PLoS One, 2012. **7**(8): p. e42264.

52. Shiozaki A, N.S., Ichikawa D, Fujiwara H, Konishi H, Komatsu S, Kubota and O.K. T, Iitaka D, Shimizu H, Nako Y, Takemoto K, Kishimoto M, Otsuji E, *Prognostic significance of p21 expression in patients with esophageal squamous cell carcinoma.* Anticancer Res, 2013. **10**(33): p. 4329-4335.

53. Solaroli, N., et al., *Identification of two active functional domains of human adenylate kinase 5.* FEBS Lett, 2009. **583**(17): p. 2872-6.

54. Vasiliou V, P.A., Petersen DR, *Role of aldehyde dehydrogenases in endogenous and xenobiotic metabolism.* Chemico-Biological Interactions, 2000. **129**(1-2): p. 1-19.

55. Ferreira, M.A., et al., *Collaborative genome-wide association analysis supports a role for ANK3 and CACNA1C in bipolar disorder.* Nat Genet, 2008. **40**(9): p. 1056-8.

56. Shiloh, G.R.a.Y., *ATM: from gene to function.* Human Molecular Genetics, 1998. **7**(10): p. 1555-1563.

57. Gvozdenovic, A., et al., *Silencing of CD44 gene expression in human 143-B osteosarcoma cells promotes metastasis of intratibial tumors in SCID mice.* PLoS One, 2013. **8**(4): p. e60329.

58. Turksen, K. and T.C. Troy, *Barriers built on claudins.* J Cell Sci, 2004. **117**(Pt 12): p. 2435-47.

59. Kominsky, S.L., et al., *Loss of the tight junction protein claudin-7 correlates with histological grade in both ductal carcinoma in situ and invasive ductal carcinoma of the breast.* Oncogene, 2003. **22**(13): p. 2021-33.

60. Jin, C., et al., *CMTM8 induces caspase-dependent and -independent apoptosis through a mitochondria-mediated pathway.* J Cell Physiol, 2007. **211**(1): p. 112-20.

61. Arantza Fariña Sarasqueta, G.I.F., Wim E Corver, Noel F de Miranda, Dina Ruano, Ronald van Eijk, Jan Oosting, Rob AEM Tollenaar, Tom van Wezel and Hans Morreau, *Integral analysis of p53 and its value as prognostic factor in sporadic colon cancer.* BMC cancer, 2013(13): p. 277-287.

62. Chun-Ming Wong, J.M.-F.L., Yick-Pang Ching, Dong-Yan Jin, and Irene Oi-lin Ng, *Eenetic and epigenetic alterations of DLC1 gene in hepatocellualar carcinoma.* Cancer Research, 2003. **63**: p. 7646-7651.

63. Davies EL, G.J., Cochrane RA, Jiang WG, Sharma AK, Nicholson RI, Mansel RE, *The Immunohistochemical Expression of Desmoplakin and its Role In Vivo in the Progression and Metastasis of Breast Cancer.* European Journal of Cancer, 1999. **35**(6): p. 902-907.

64. Yang, L., et al., *Desmoplakin acts as a tumor suppressor by inhibition of the Wnt/beta-catenin signaling pathway in human lung cancer.* Carcinogenesis, 2012. **33**(10): p. 1863-70.

65. Chun, M.G. and D. Hanahan, *Genetic deletion of the desmosomal component desmoplakin promotes tumor microinvasion in a mouse model of pancreatic neuroendocrine carcinogenesis.* PLoS Genet, 2010. **6**(9): p. e1001120.

66. Pang, H., et al., *Epidermal growth factor suppresses induction by progestin of the adhesion protein desmoplakin in T47D breast cancer cells.* Breast Cancer Res, 2004. **6**(3): p. R239-45.

67. Chaudhry SS, G.J., Baldock C, Dixon J, Rock MJ, Skinner GC, Steel KP, *Mutation of the gene encoding fibrillin-2 results in syndactyly in mice.* Human Molecular Genetics, 2001. **10**(8): p. 835-843.

68. Zheng, H., et al., *Essential role of Fbxl14 ubiquitin ligase in regulation of vertebrate axis formation through modulating Mkp3 level.* Cell Res, 2012. **22**(5): p. 936-40.

69. Vinas-Castells, R., et al., *The hypoxia-controlled FBXL14 ubiquitin ligase targets SNAIL1 for proteasome degradation.* J Biol Chem, 2010. **285**(6): p. 3794-805.

70. Akiyama, Y., et al., *GATA-4 and GATA-5 Transcription Factor Genes and Potential Downstream Antitumor Target Genes Are Epigenetically Silenced in Colorectal and Gastric Cancer.* Molecular and Cellular Biology, 2003. **23**(23): p. 8429-8439.

71. Dong X, L.Y., Chang P, Tang H, Hess KR, Abbruzzese JL, Li D, *Glucose Metabolism Gene Variants Modulate the Risk of Pancreatic Cancer.* Cancer Prevention Research, 2011. **4**: p. 758-766.

72. Fang, R., et al., *MicroRNA-143 (miR-143) regulates cancer glycolysis via targeting hexokinase 2 gene.* J Biol Chem, 2012. **287**(27): p. 23227-35.

73. Griffith, L.G. and M.A. Swartz, *Capturing complex 3D tissue physiology in vitro.* Nat Rev Mol Cell Biol, 2006. **7**(3): p. 211-24.

74. Regel, I., et al., *IGFBP3 impedes aggressive growth of pediatric liver cancer and is epigenetically silenced in vascular invasive and metastatic tumors.* Molecular cancer, 2012. **11**: p. 9.

75. Dhanoa BS, C.T., Satish AG, Bruford EA, Friedman JS, *Update on the Kelch-like (KLHL) gene family.* Human Genomics, 2013. **7**(13).

76. Iyer, S.V., et al., *Understanding the role of keratins 8 and 18 in neoplastic potential of breast cancer derived cell lines.* PLoS One, 2013. **8**(1): p. e53532.

77. Oshima, R., *Apoptosis and keratin intermediate filaments.* Cell Death and Differentiation, 2002. **9**: p. 486-492.

78. Somiari, S.B., et al., *Circulating MMP2 and MMP9 in breast cancer -- potential role in classification of patients into low risk, high risk, benign disease and breast cancer categories.* International journal of cancer. Journal international du cancer, 2006. **119**(6): p. 1403-11.

79. Sakurai, T., *The role of NrCAM in neural development and disorders--beyond a simple glue in the brain.* Mol Cell Neurosci, 2012. **49**(3): p. 351-63.

80. Martin, *Loss of occludin leads to the progression of human breast cancer.* International Journal of Molecular Medicine, 2010. **26**(5).

81. Varela, I., et al., *Exome sequencing identifies frequent mutation of the SWI/SNF complex gene PBRM1 in renal carcinoma.* Nature, 2011. **469**(7331): p. 539-42.

82. Sugrue, O.a., *Characterization of Pinin, A Novel Protein Associated with the Desmosome-Intermediate Filament Complex.* The Journal of Cell Biology, 1996. **135**(November 4): p. 1027-1042.

83. Araki, K.M.Y.a.M., *Tumor suppressor PTEN: modulator of cell signaling, growth, migration and apoptosis.* Signal Transduction and Cellular Organization, 2001. **114**: p. 2375-2382.

84. Xinyi Wu, k.S., Mehran S, Young E. Whang and Charles L. Sawyers, *The PTEN MMAC1 tumor suppressor phosphatase functions as a negative regulator of the phosphoinositide 3-kinase Akt pathway.* Proc. Natl. Acad. Sci, 1998. **95**: p. 15587-15591.

85. Siraj, A.K., et al., *Demethylation of TMS1 gene sensitizes thyroid cancer cells to TRAIL-induced apoptosis.* The Journal of clinical endocrinology and metabolism, 2011. **96**(1): p. E215-24.

86. Kehlen A, H.M., Menge K, Gans K, Immel UD, Hoang-Vu C, Klonisch T, Demuth and L. Hu, *Role of glutaminyl cyclases in thyroid carcinomas.* Endocr Relat cancer 2012. **20**(1): p. 79-90.

87. Pierga, J.Y., et al., *Circulating tumor cells and brain metastasis outcome in patients with HER2-positive breast cancer: the LANDSCAPE trial.* Ann Oncol, 2013. **24**(12): p. 2999-3004.

88. Gimenez-Roqueplo, A.P., et al., *The R22X mutation of the SDHD gene in hereditary paraganglioma abolishes the enzymatic activity of complex II in the mitochondrial respiratory chain and activates the hypoxia pathway.* Am J Hum Genet, 2001. **69**(6): p. 1186-97.

89. Chau BN, D.R., Saunders MA, Cheng C, Chang AN, Warrener P, Bradshaw J, and C.M. Linsley PS, *Identification of SULF2 as a Novel Transcriptional Target of p53 by Use of Integrated Genomic Analyses*

*.* Cancer Research, 2009. **69**: p. 1368-1374.

90. Li, W. and R.A. Cornell, *Redundant activities of Tfap2a and Tfap2c are required for neural crest induction and development of other non-neural ectoderm derivatives in zebrafish embryos.* Dev Biol, 2007. **304**(1): p. 338-54.

91. D'Atri, F., F. Nadalutti, and S. Citi, *Evidence for a functional interaction between cingulin and ZO-1 in cultured cells.* J Biol Chem, 2002. **277**(31): p. 27757-64.

92. Gery, S. and H.P. Koeffler, *Repression of the TMEFF2 Promoter by c-Myc.* Journal of Molecular Biology, 2003. **328**(5): p. 977-983.

93. Miloloza A, R.M., Nellist M, Halley D, Bernaschek G, Hengstschläger M, *The TSC1 gene product hamartin, negatively regulates cell proliferation.* Human Molecular Genetics, 2000. **9**(12): p. 1721-1727.

94. Jose M. Arencibia, S.M., Fransisco J. Perez-Rodriguez and Ana Bonnin, *Gene expression profiling reveals overexpression of TSPAN13 in prostate cancer.* International journal of Oncology, 2009. **34**: p. 457-463.

95. Todd SC, D.V., Levy S, *Sequences and expression of six new members of the tetraspanin/TM4SF family.* 1998: p. 101-104.

96. Bilguvar K, T.N., Ozkara C, Tuysuz B, Bakircioglu M, Choi M, Delil S,, B.J. Caglayan AO, Erturk O, Yalcinkaya C, Karacorlu M, Dincer A, Johnson, and M.S. MH, Chandra SS, Louvi A, Boggon TJ, Lifton RP, Horwich AL, Gunel M, *Recessive loss of function of the neuronal ubiquitinhydrolase UCHL1 leads to early-onset progressive neurodegeneration.* PNAS, 2012. **110**(9): p. 3489-3494.

97. Lancet D, S.G., Golan Y, and Rinon A, *Gene Trends: On Muscle, Fat, and Brain.* Genetic Engineering and Biotechnology News, 2013(Dec 16).
