# Supplementary figures and images for: The GALNT9, BNC1 and CCDC8 genes are frequently epigenetically dysregulated in breast tumours that metastasise to the brain
Source: Clin Epigenetics. 2015 May 27;7(1):57. doi: 10.1186/s13148-015-0089-x (PMC4457099; doi:10.1186/s13148-015-0089-x)

### Supplementary Figure 3

## BNC1 CoBRA analysis

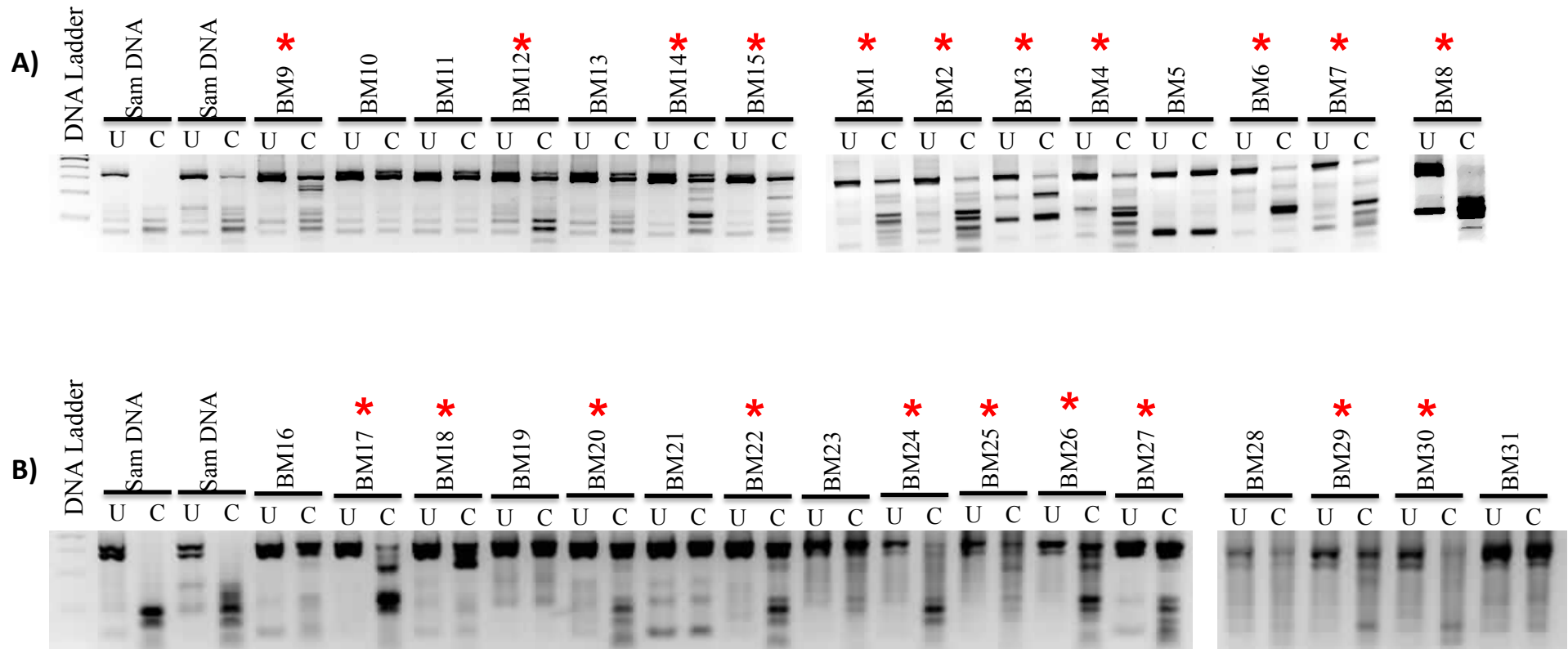

## CCDC8 CoBRA analysis

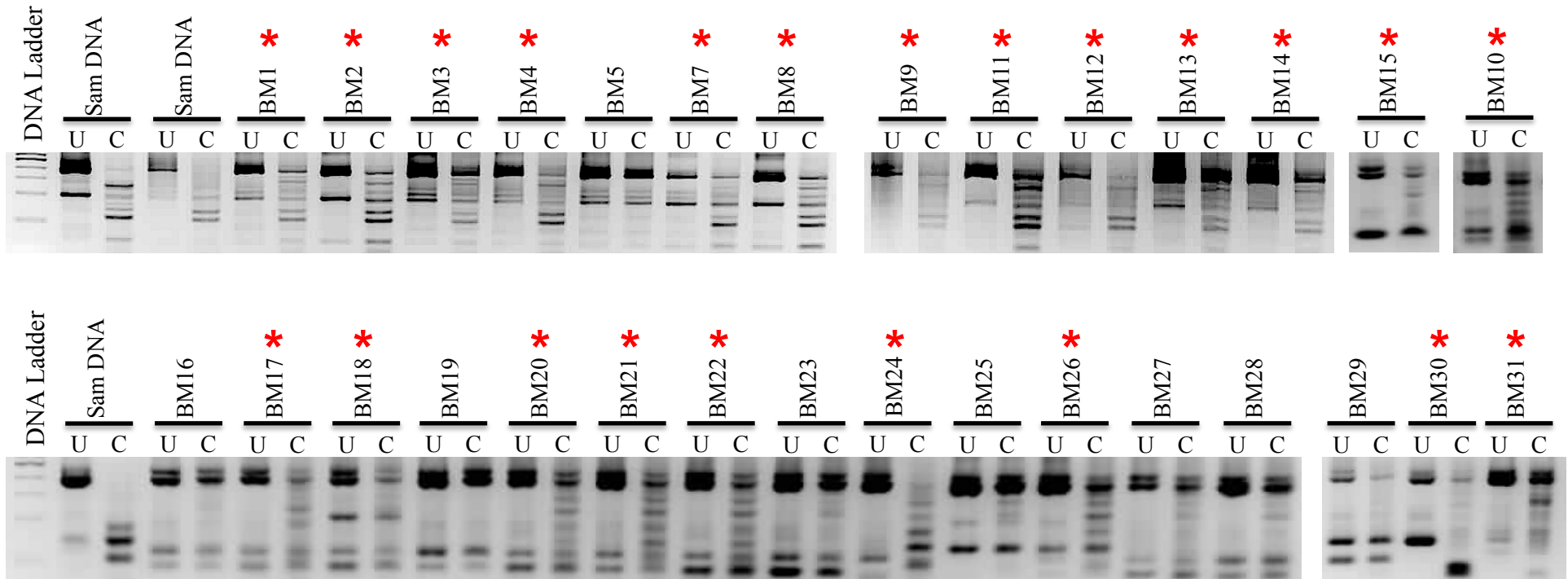

***GALT*N9 CoBRA analysis**

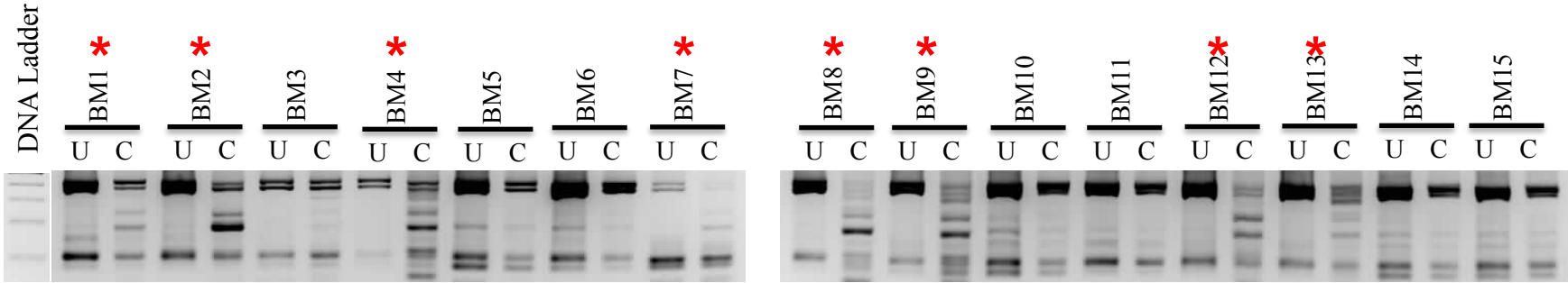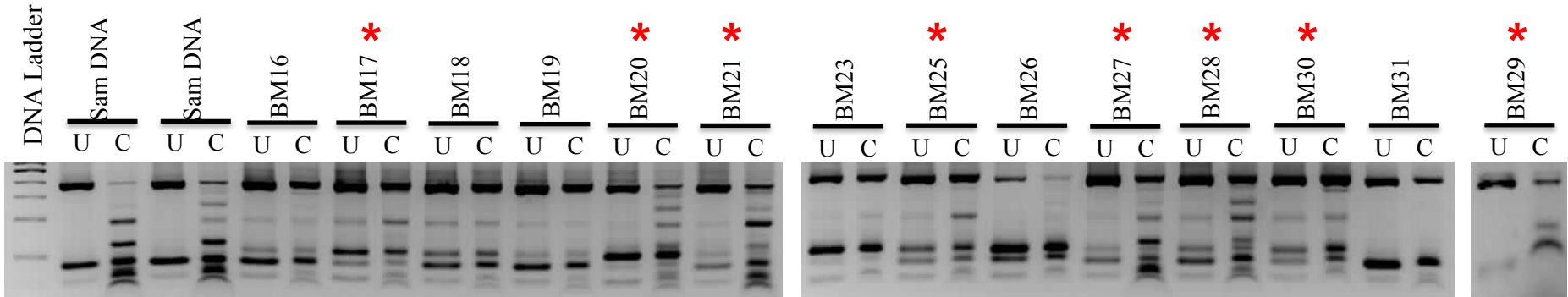

Supplement: Additional file 7: Figure S3. — Methylation analysis of BNC1, CCDC8 and GALNT9 in Breast to brain metastases. Up to 31 brain metastases (BM) were analysed by CoBRA, small, digested PCR products in the Bstu1 cut (C) lane compared to the undigested (U) lane indicates promoter methylation in a sample (SAM DNA: genomic DNA treated with S-Adenosyl methionine and DNA methyltransferase as a positive control). [file 13148_2015_89_MOESM7_ESM.pdf]

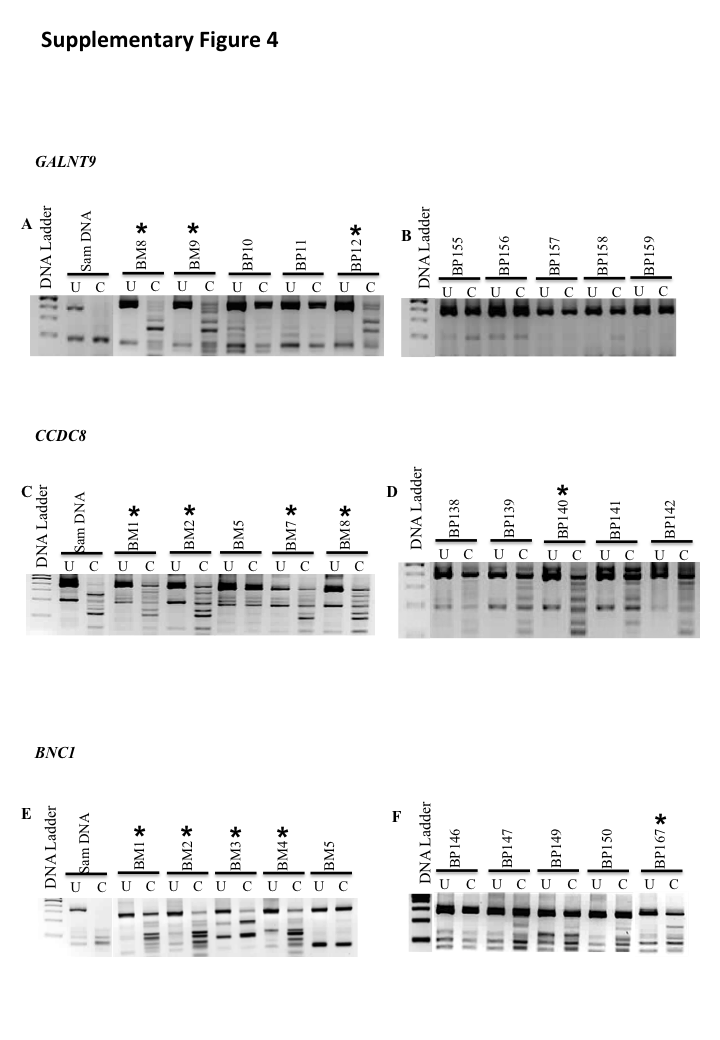

Supplement: Additional file 8: Figure S4. — Methylation status of GALNT9, CCDC8 and BNC1 in metastatic brain tumours from primary breast tumours and a cohort of unrelated primary breast tumours. (A) GALNT9 was frequently methylated in metastatic brain tumours (55 %) and (B) was NOT methylated in any of the primary tumours; (C) CCDC8 was frequently methylated in metastatic brain tumours (73 %) and (D) infrequently methylated in primary breast tumours (40 %). (E) BNC1 is frequently methylated in metastatic brain tumours 68 % and (F) infrequently methylated in a cohort of unrelated primary breast tumours (17 %). We have determined that a significant proportion of the promoters within the tumour sample are methylated if there are clearly observed digest products following restriction analysis BM: Brain metastases, BP: Primary breast tumours, U: Uncut/control sample, C: cut by restriction enzyme, *: methylated samples). [file 13148_2015_89_MOESM8_ESM.tiff]

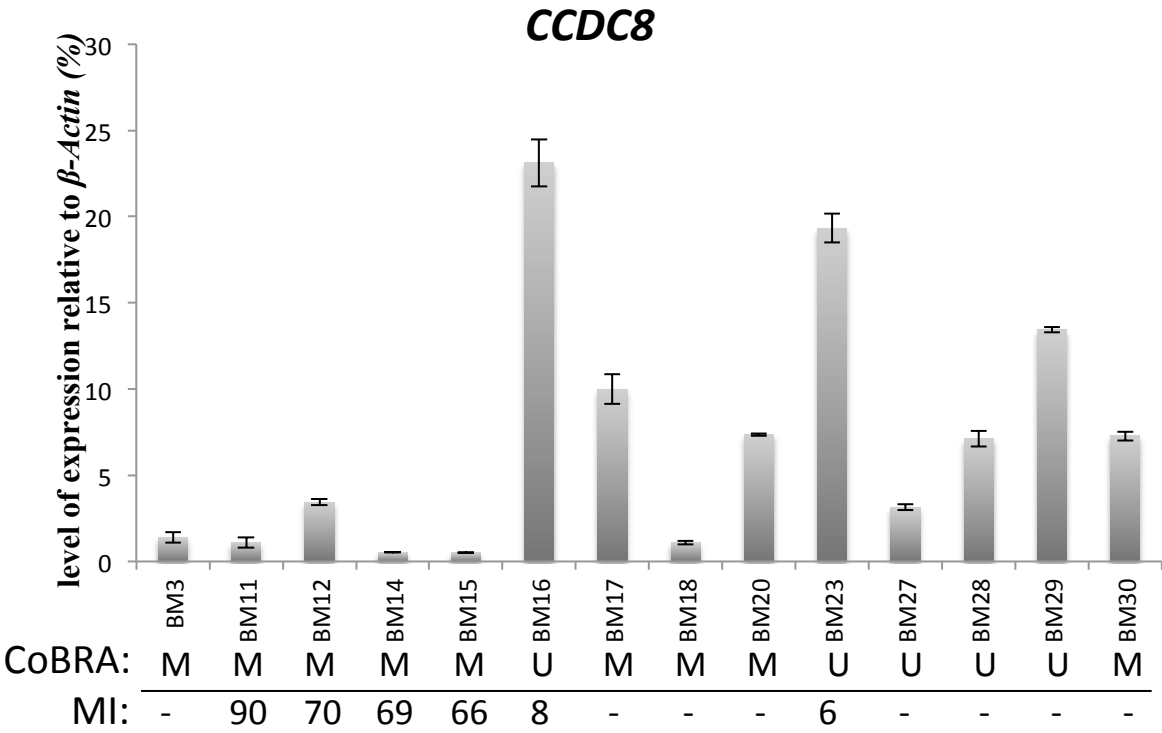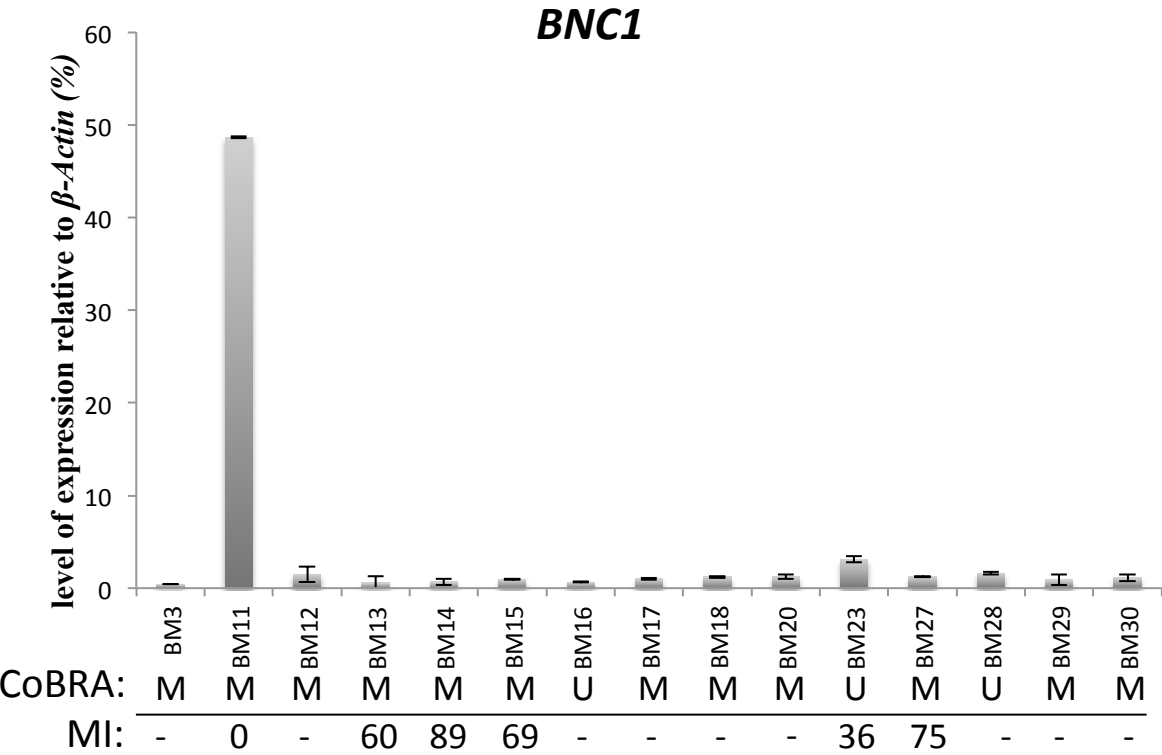

GALNT9

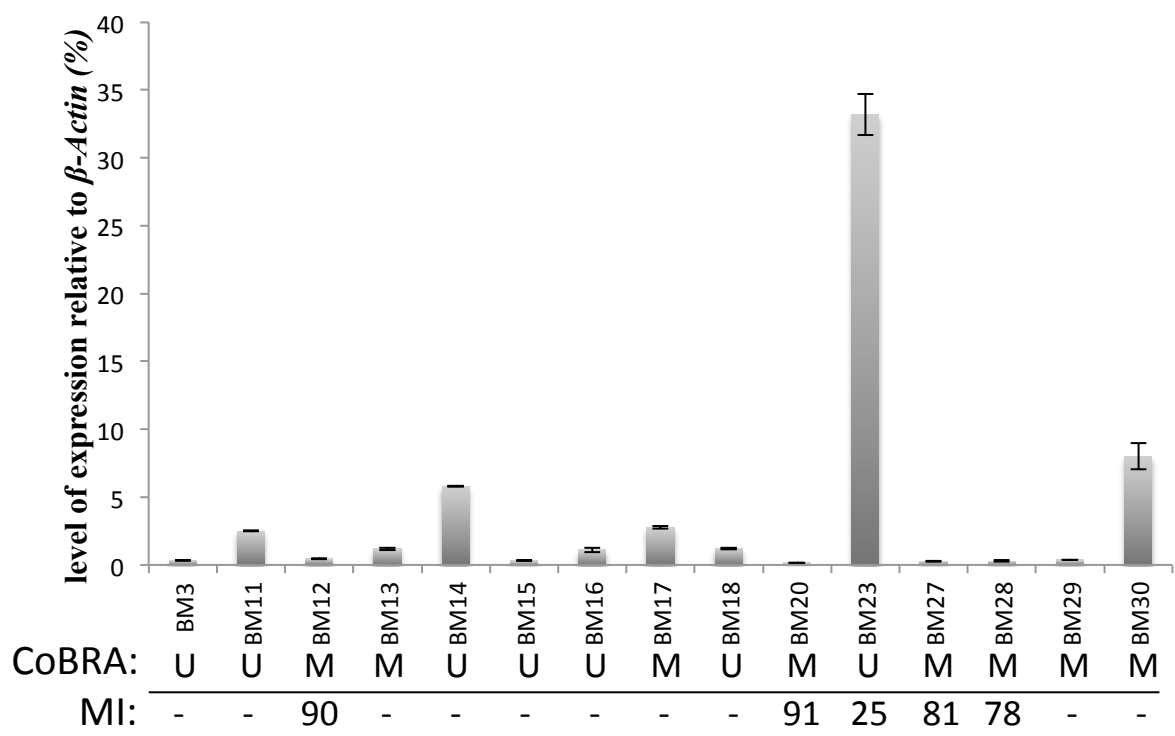

Supplement: Additional file 10: Figure S6. — Expression levels of BNC1. CCDC8 and GALNT9 in all tumours analysed. The expression level of each gene was quantified in relation to the expression of β-actin. Below each bar is the methylation status of each CpG island as determined by CoBRA and sequencing of individual alleles (MI) (BM: Brain Metastasis, MI: Methylation index, M: Methylated, U: Unmethylated, -: analysis was not done). [file 13148_2015_89_MOESM10_ESM.pdf]

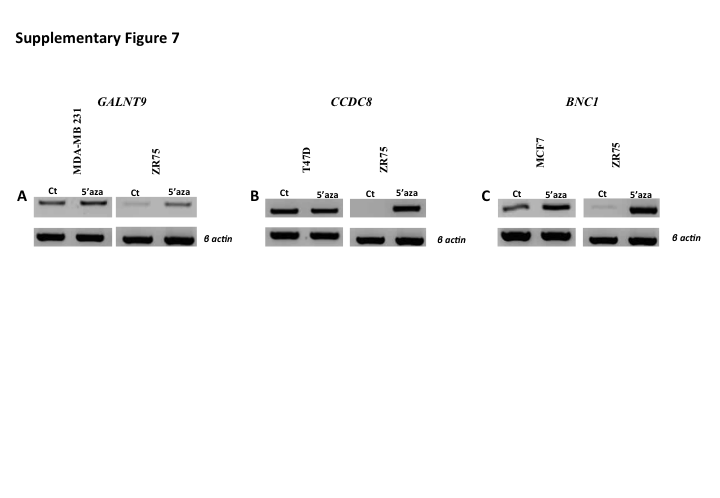

Supplement: Additional file 11: Figure S7. — Global demethylation resulted in the re-expression of GALNT9, CCDC8 and BNC1 in breast cancer cell lines. Reverse transcription PCR (RT-PCR) showed that treatment of breast cancer cell lines with 5- 2-deoxycytidine (5-AZA-dC), an inhibitor of DNA methyltransferase enzymes, resulted in re-expression of (A) GALNT9, (B) CCDC8 or (C) BNC1 in the breast cancer cell line ZR75. For comparison, endogenous expression is shown in (A) MDA-MD231, (B) T47D and (C) MCF7, these, expressing, cell lines were used in our in vitro knock down experiments. [file 13148_2015_89_MOESM11_ESM.tiff]

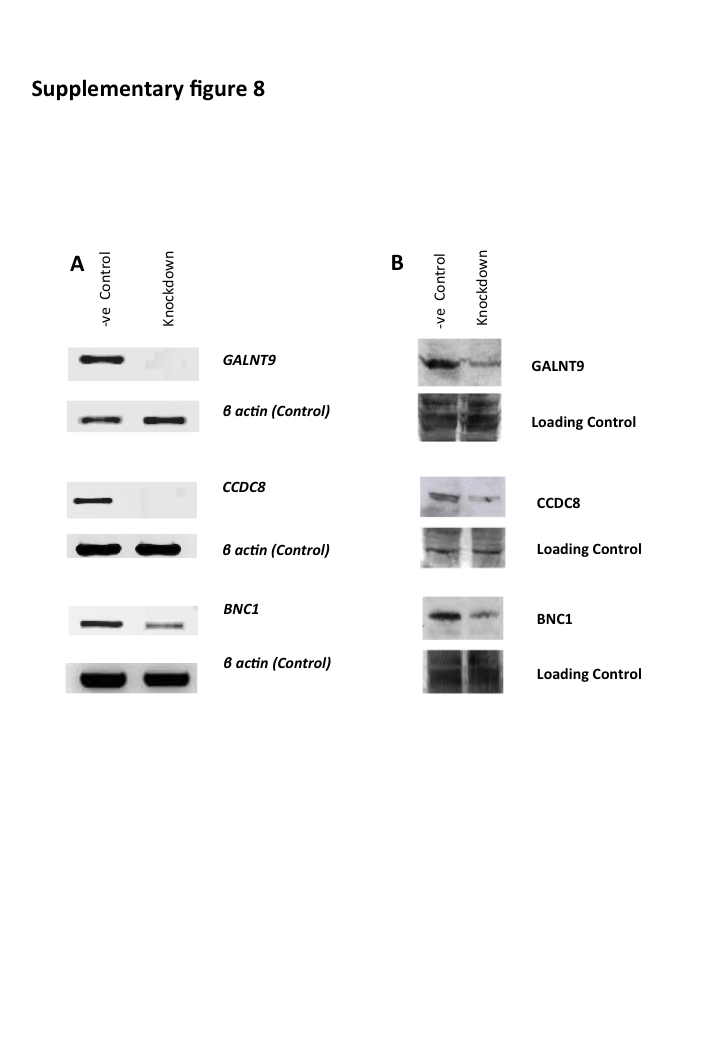

Supplement: Additional file 12: Figure S8. — Knockdown of GALNT9, CCDC8, and BNC1 in breast cancer cell lines is confirmed by Reverse transcription (RT) PCR and western blot. (A) RT-PCR of GALNT9, CCDC8, and BNC1 transcripts in breast cancer cell lines (MDA-MD231, T47D and MCF7 respectively) following siRNA knockdown compared to transfection with a control siRNA and (B) western blot of GALNT9, CCDC8, and BNC1 proteins to confirm their knockdown in each respective cell line. 70 μg of protein was loaded in each lane. Equal loading was confirmed by staining total protein with India ink. [file 13148_2015_89_MOESM12_ESM.tiff]
