# Supplementary material for: The GALNT9, BNC1 and CCDC8 genes are frequently epigenetically dysregulated in breast tumours that metastasise to the brain
Source: Clin Epigenetics. 2015 May 27;7(1):57. doi: 10.1186/s13148-015-0089-x (PMC4457099; doi:10.1186/s13148-015-0089-x)

Supplementary Figure 5 a

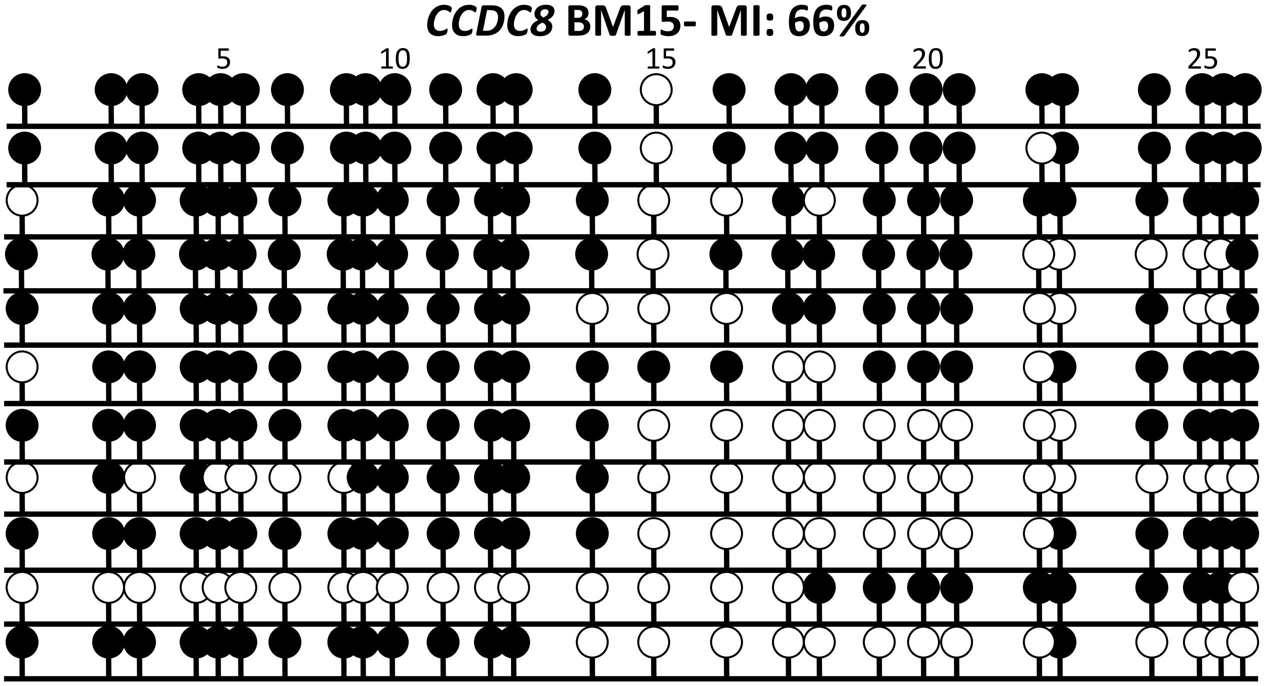

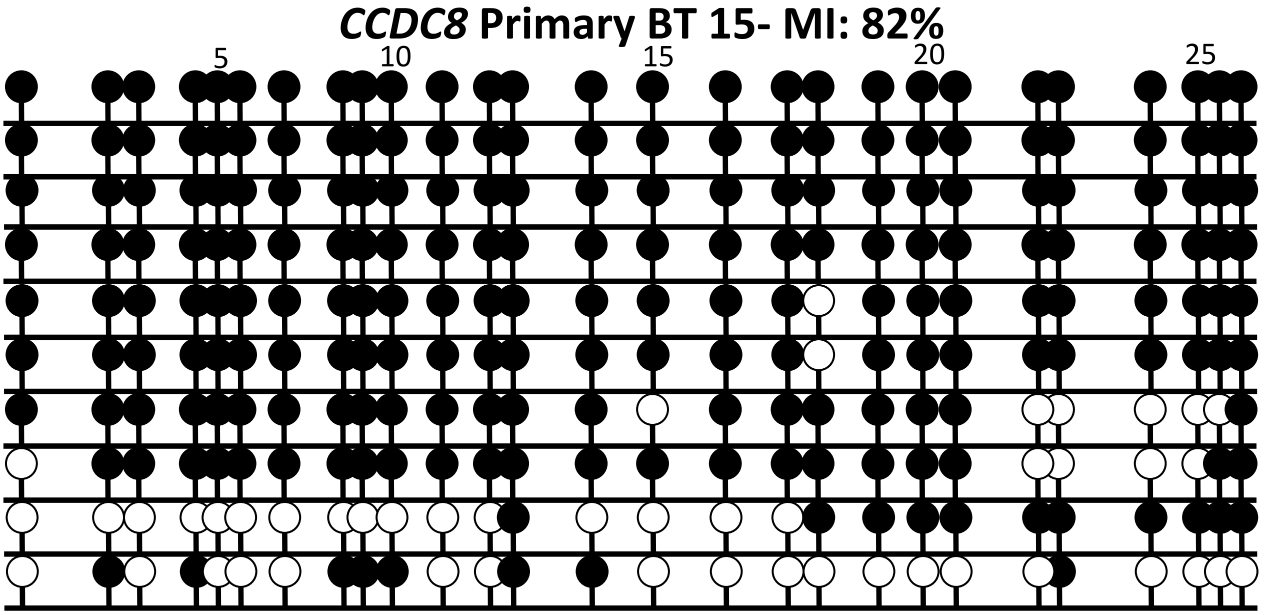

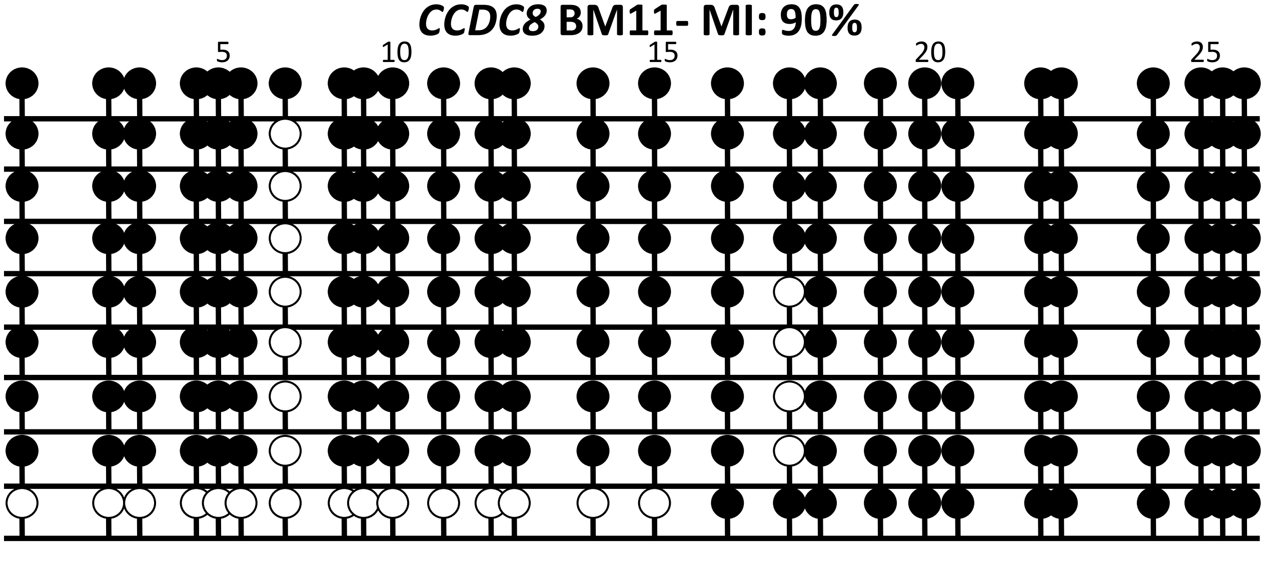

***CCDC8* Primary BT 11- MI: 74%**

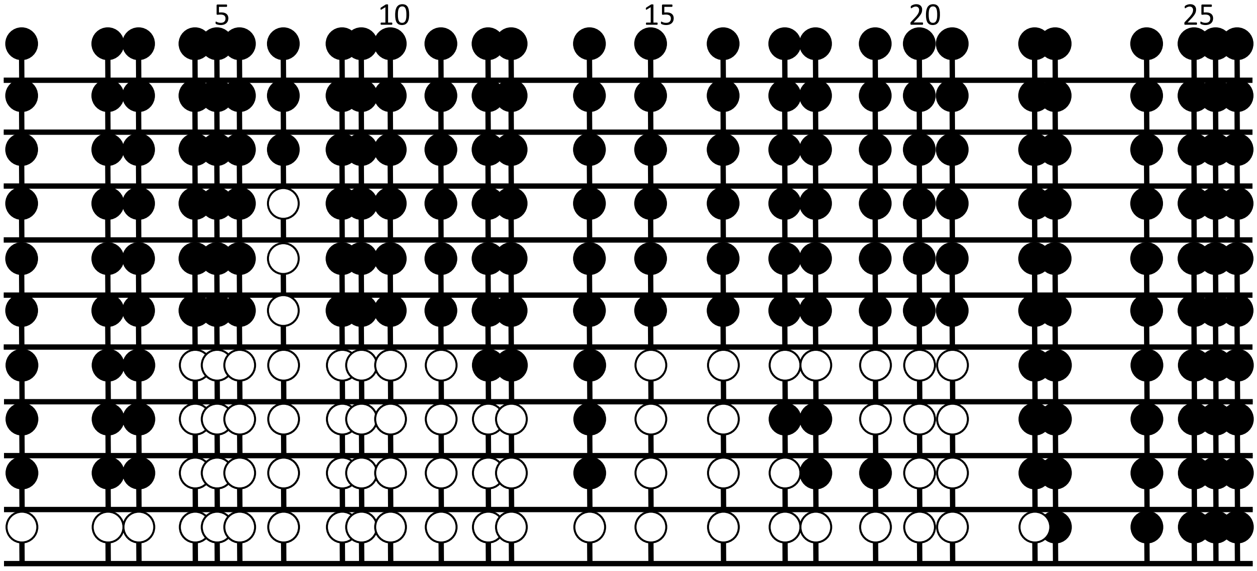

***CCDC8* BM12- MI:70%**

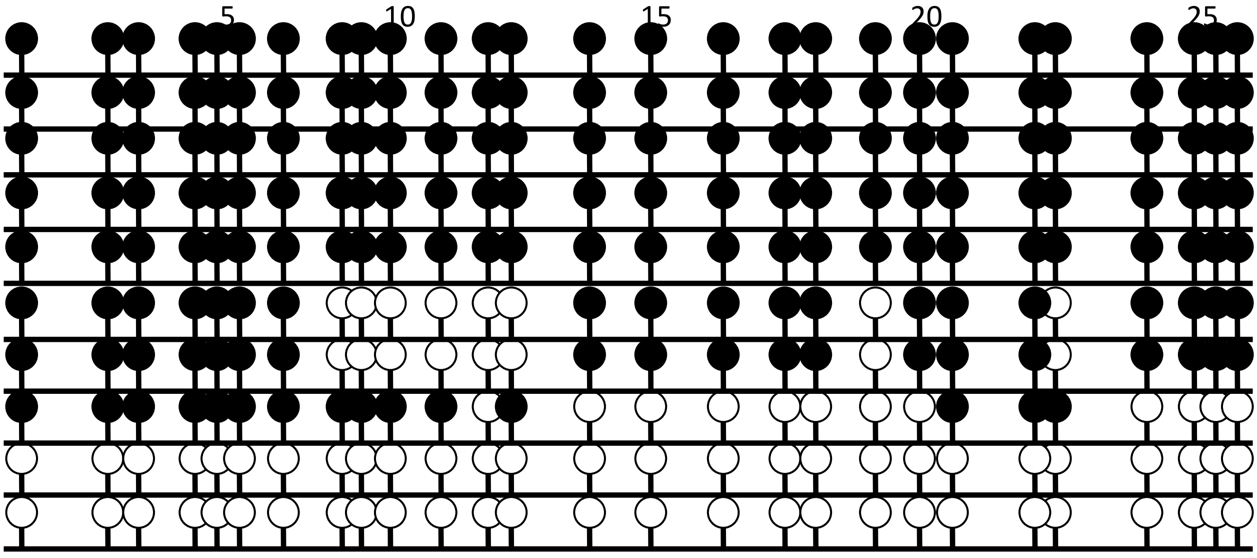

***CCDC8* BM14- MI: 69%**

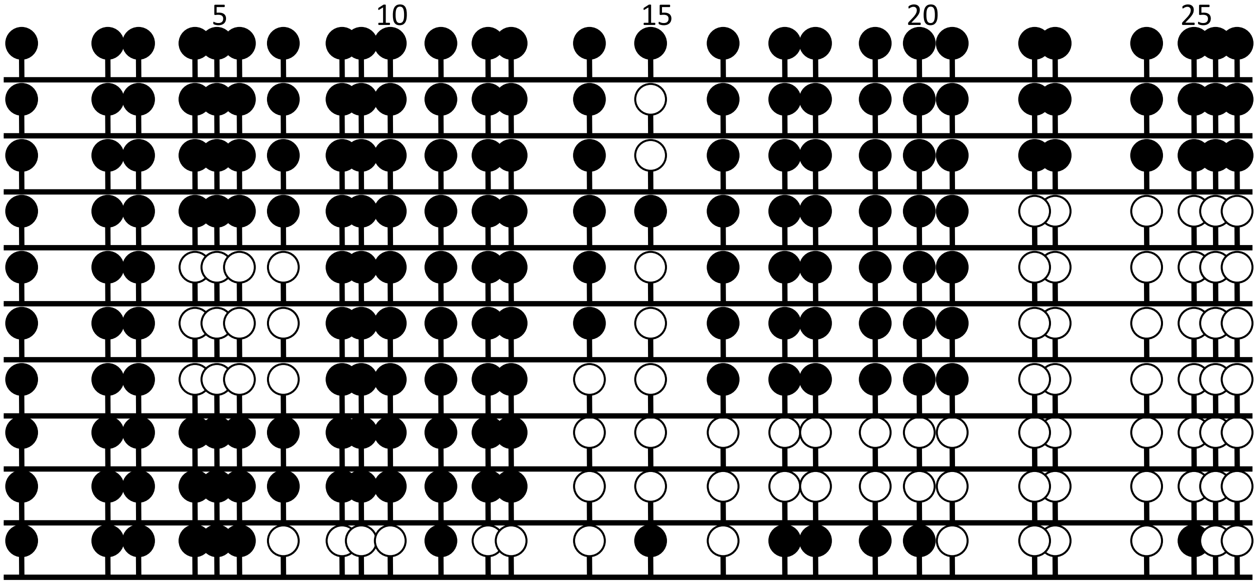

***CCDC8* BM16- MI: 8%**

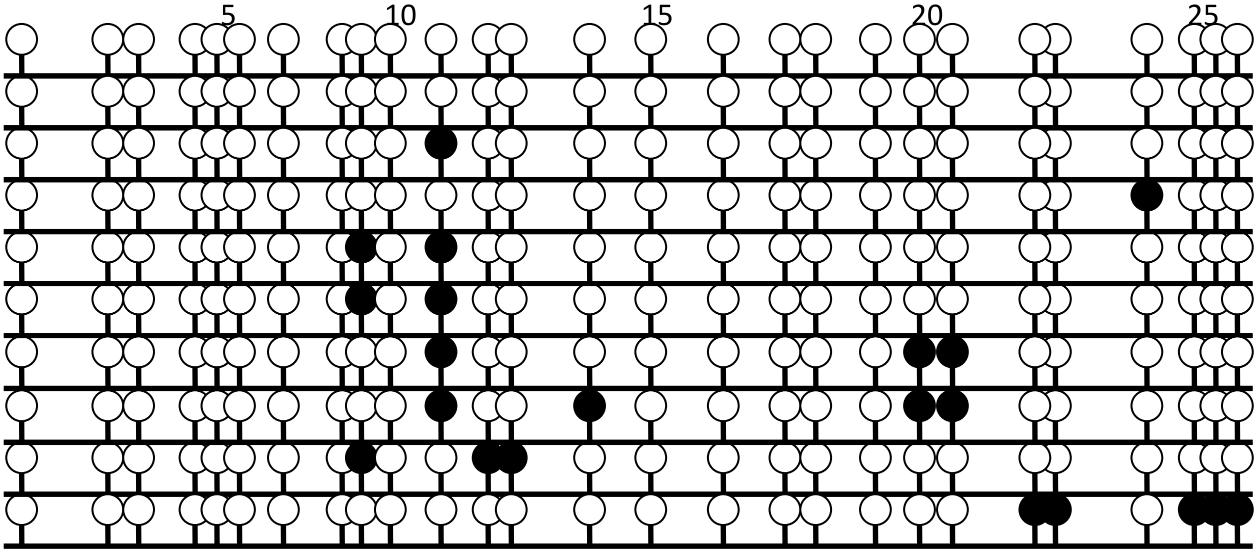

***CCDC8* BM23- MI: 6%**

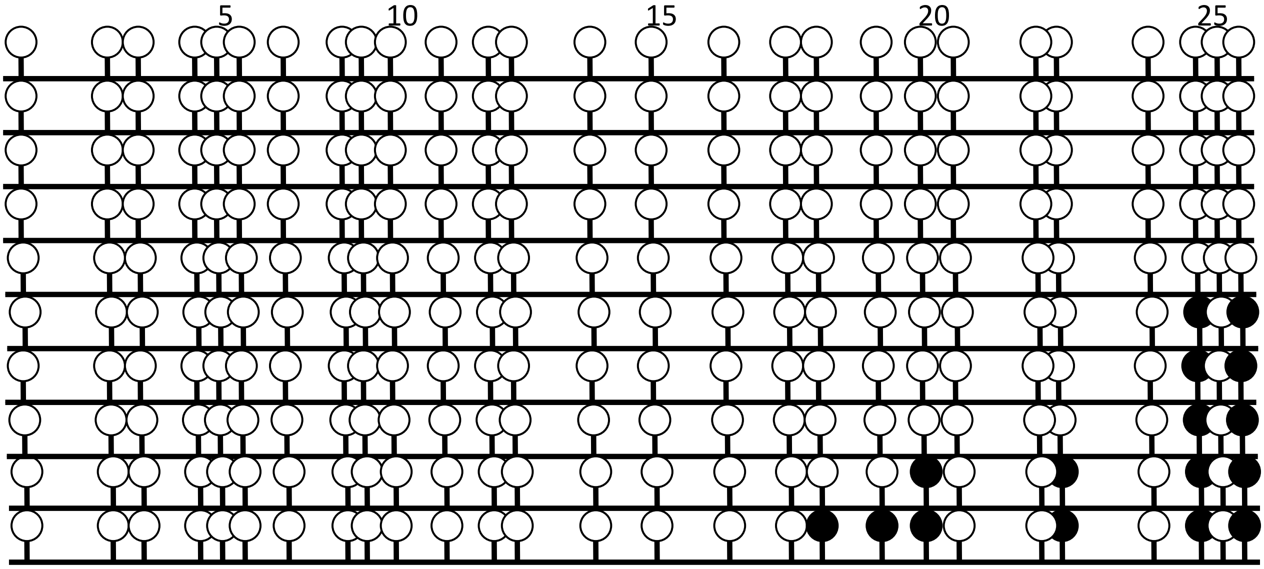

Supplementary Figure 5 B

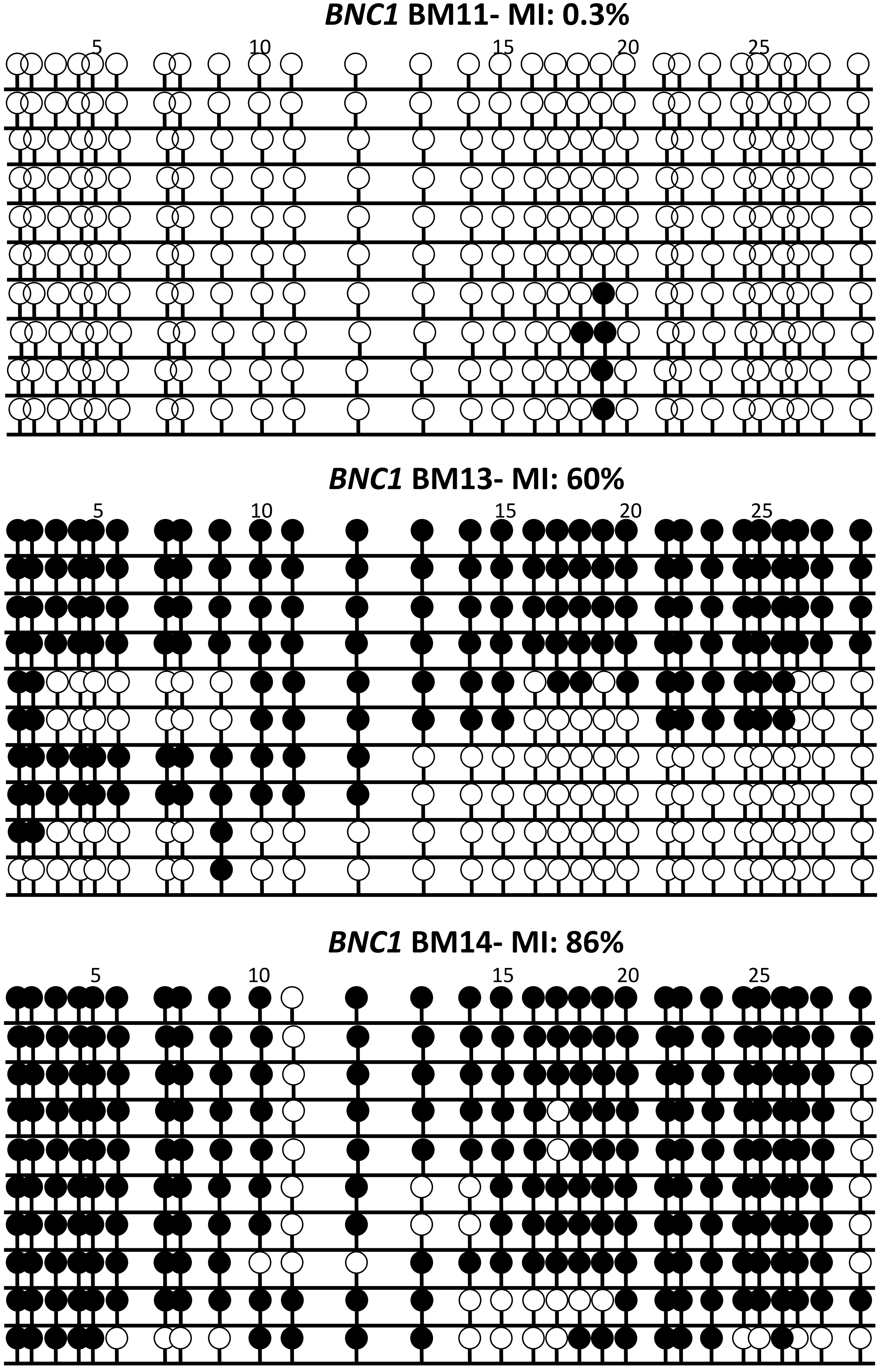

BNC1 BM15- MI:69%

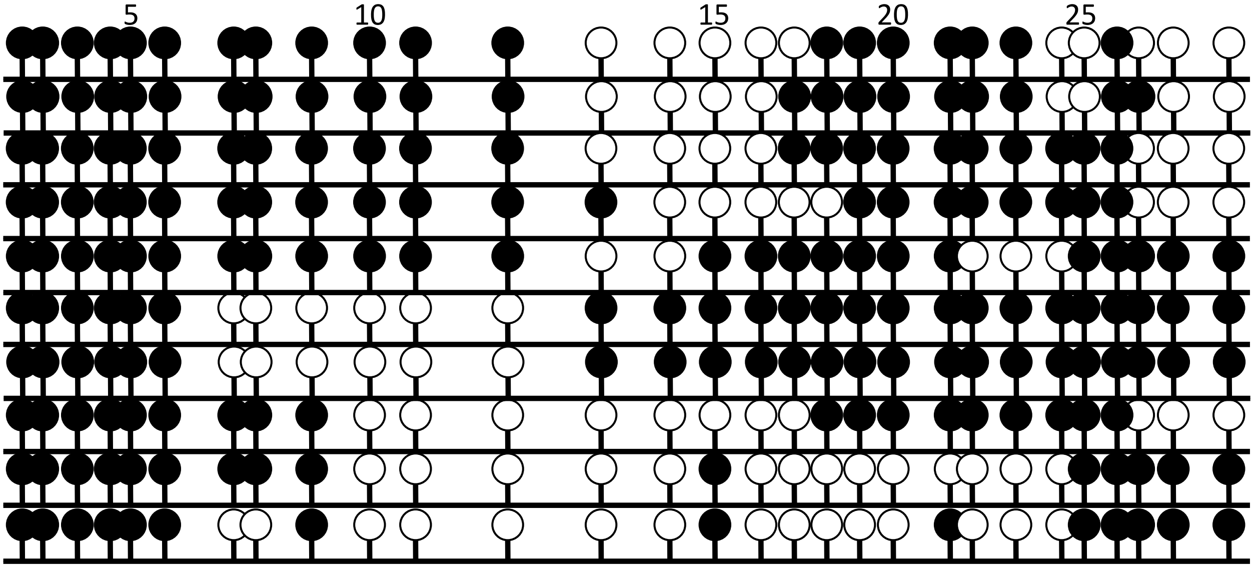

BNC1 BM23- MI: 36%

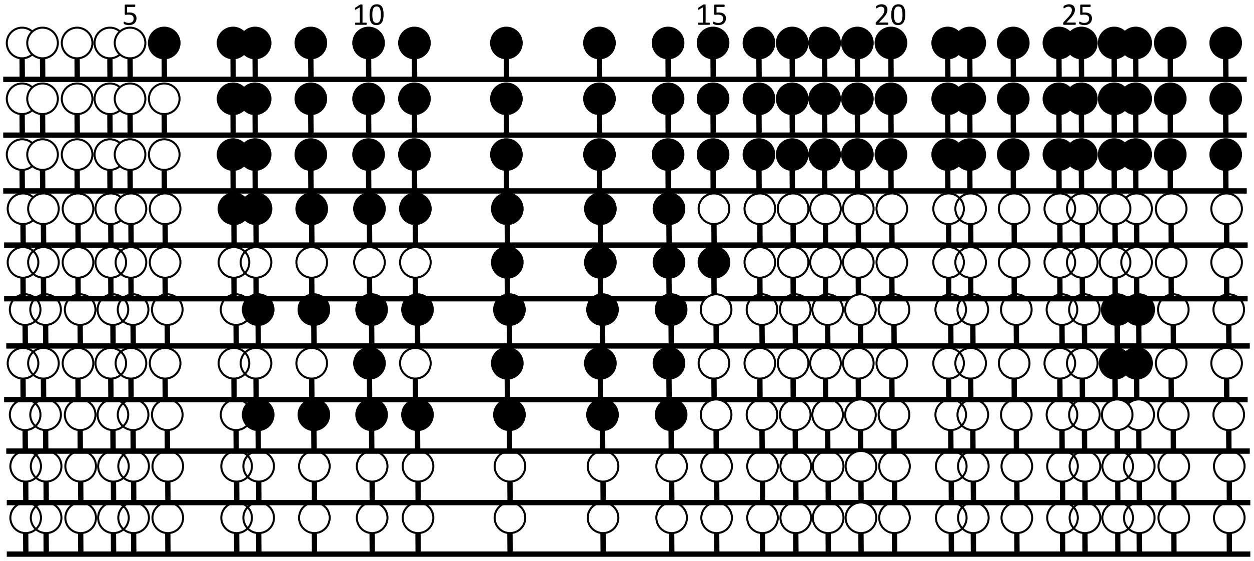

BNC1 BM27- MI: 75%

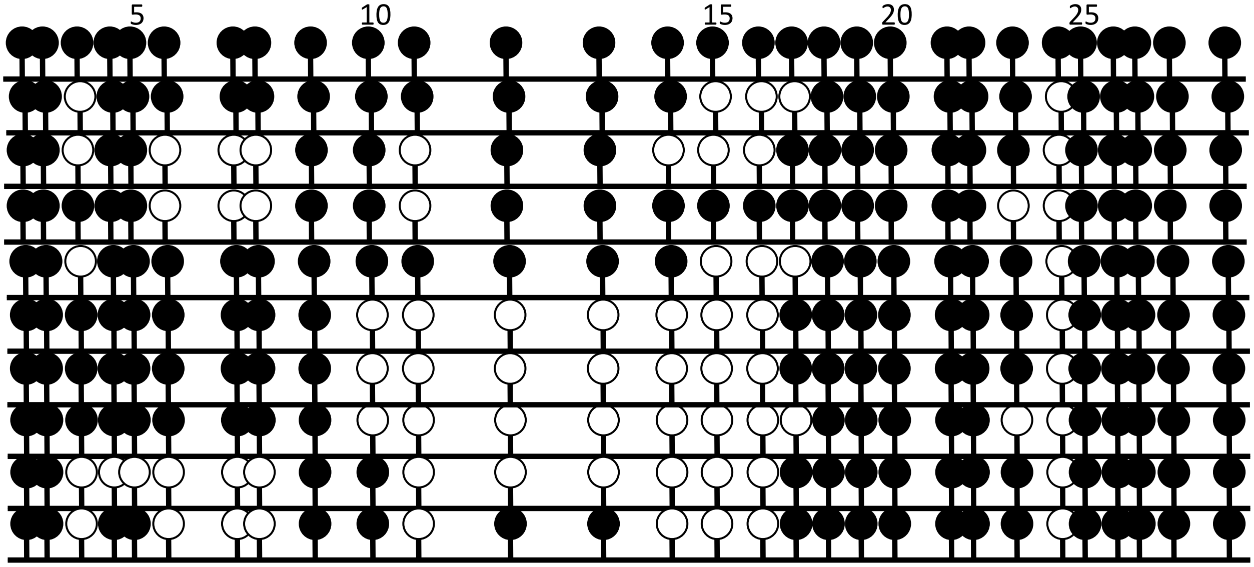

Supplementary Figure 5 C

GALNT9 BM12- MI: 90%

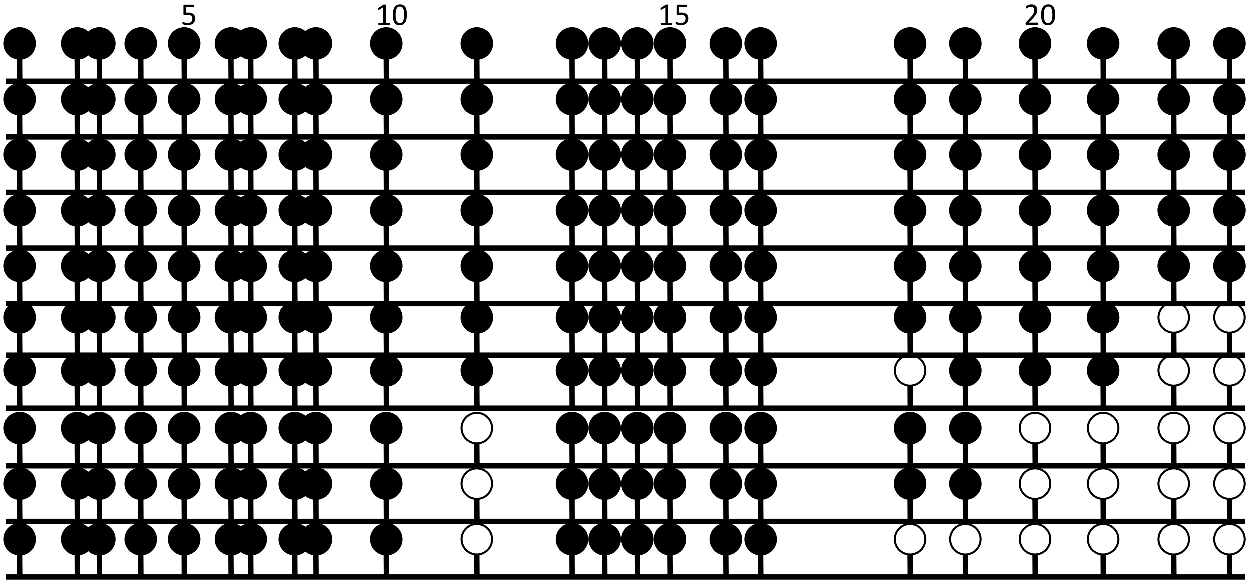

GALNT9 BM20- MI: 91%

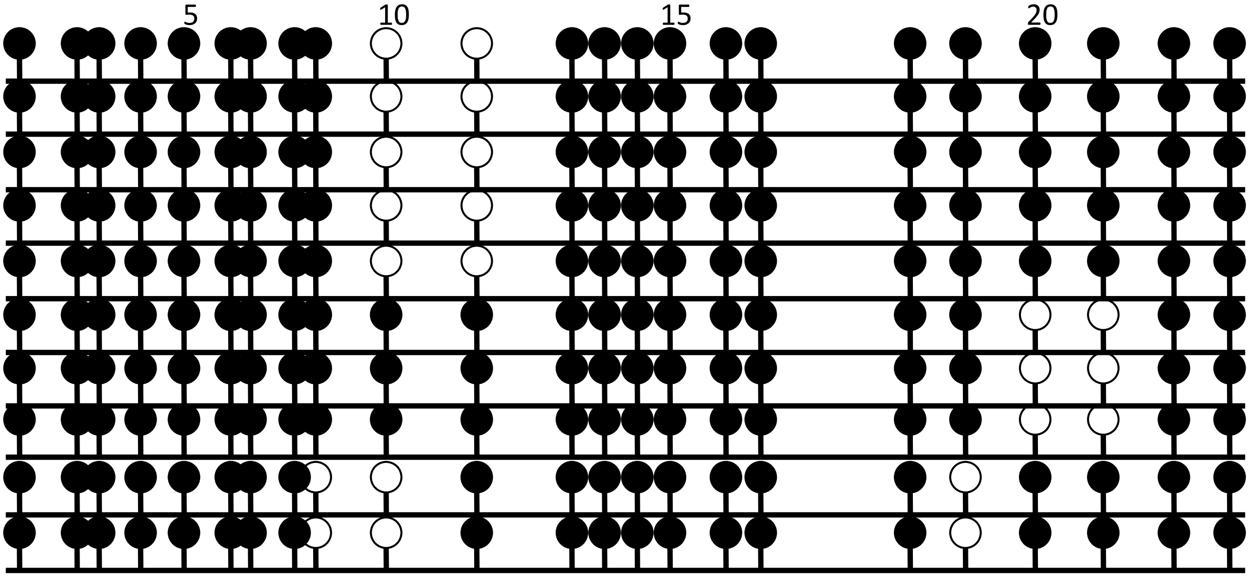

GALNT9 BM23- MI: 25%

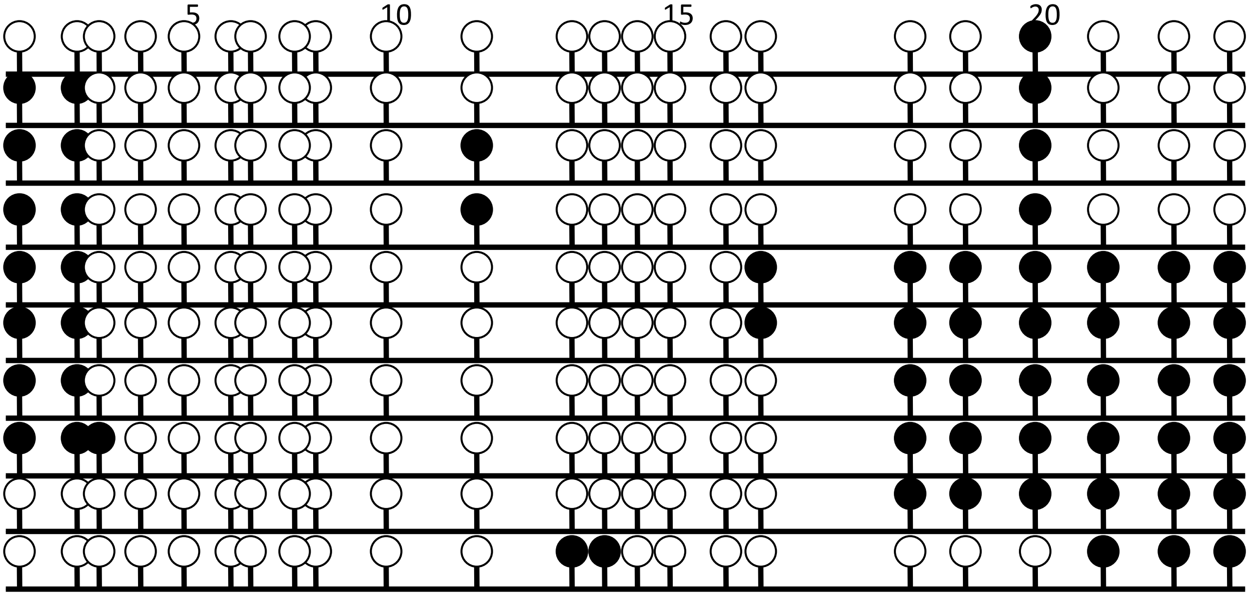

***GALNT9* BM27- MI: 81%**

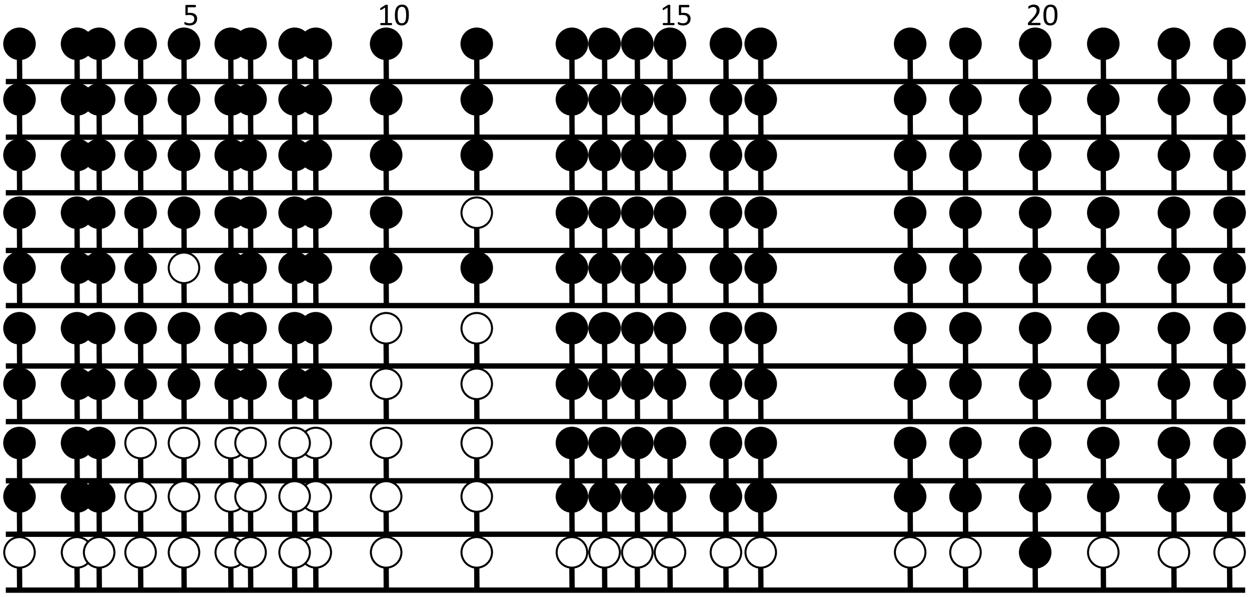

***GALNT9* BM28- MI: 78%**

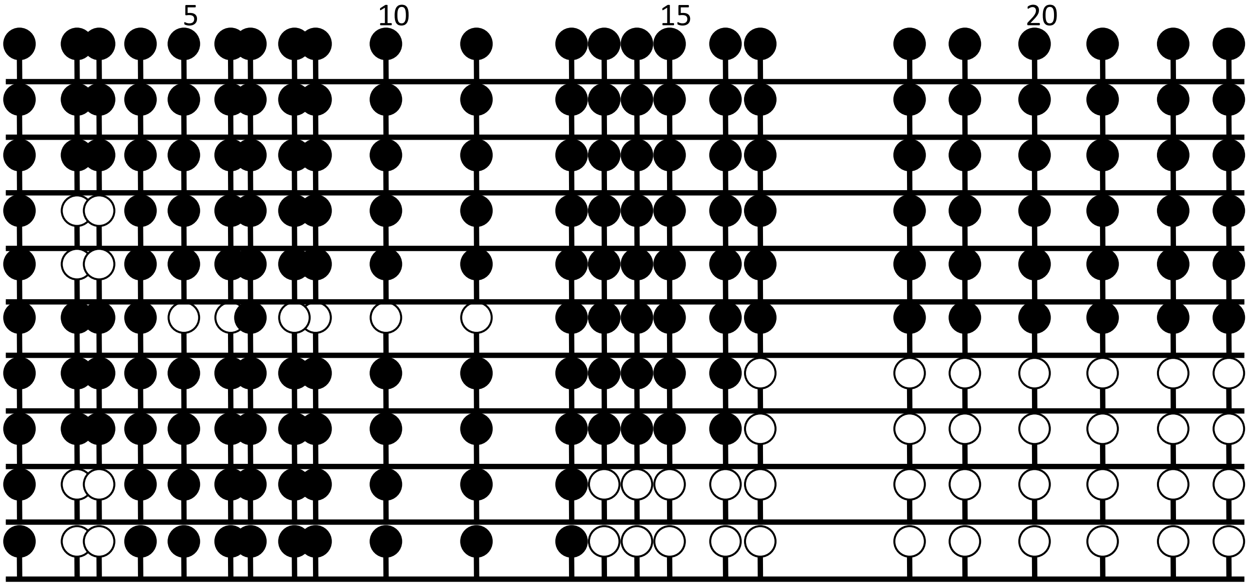

Supplement: Additional file 9: Figure S5. — Bisulphite sequencing of individual alleles form tumours. Tumours were analysed by cloning and sequencing bisulphite-PCR products to determine the extent of methylation within the region analysed by CoBRA. 10 clones/alleles were sequenced for each tumour and the methylation index (MI) for each tumour determined. (A) Tumours that were determined to have significant CCDC8 promoter methylation by CoBRA (BM11, BM15, BM12 and BM14) had methylation indices ranging from 66 %-90 %. The corresponding primary breast tumours for BM11 and BM15 were also analyses these both had correspondingly high MIs (82 % and 74 % respectively). Tumours that had no evidence of CCDC8 promoter region methylation by CoBRA analysis (BM16, BM23) had low MIs (8 % and 6 % respectively). (B) Tumours that were determined to have significant BNC1 promoter methylation by CoBRA (BM13, BM14, BM15 and BM27) had methylation indices ranging from 60 %-86 %. Tumours that had no evidence of BNC1 promoter region methylation by CoBRA analysis (BM11, BM23) had low MIs (0 % and 36 % respectively). (C) Tumours that were determined to have significant GALNT9 promoter methylation by CoBRA (BM12, BM20, BM27 and BM28) had methylation indices ranging from 78 %-91 %. Tumour BM23 that had no evidence of GALNT9 promoter region methylation by CoBRA analysis had a low MI (25 %). Each circle represents a CpG island, those shaded black are methylated. MI is defended as the total number of methylated CpG dinucleotides given as a percentage of all CpGs analysed. [file 13148_2015_89_MOESM9_ESM.pdf]
